# Soft, skin-interfaced microfluidic systems with integrated immunoassays, fluorometric sensors, and impedance measurement capabilities

**DOI:** 10.1073/pnas.2012700117

**Published:** 2020-10-26

**Authors:** Sungbong Kim, Boram Lee, Jonathan T. Reeder, Seon Hee Seo, Sung-Uk Lee, Aurélie Hourlier-Fargette, Joonchul Shin, Yurina Sekine, Hyoyoung Jeong, Yong Suk Oh, Alexander J. Aranyosi, Stephen P. Lee, Jeffrey B. Model, Geumbee Lee, Min-Ho Seo, Sung Soo Kwak, Seongbin Jo, Gyungmin Park, Sunghyun Han, Inkyu Park, Hyo-Il Jung, Roozbeh Ghaffari, Jahyun Koo, Paul V. Braun, John A. Rogers

**Affiliations:** ^a^Querrey Simpson Institute for Bioelectronics, Northwestern University, Evanston, IL 60208;; ^b^Department of Materials Science and Engineering, University of Illinois at Urbana–Champaign, Urbana, IL 61801;; ^c^Materials Research Laboratory, University of Illinois at Urbana–Champaign, Urbana, IL 61801;; ^d^Department of Medicine, Konkuk University, Seoul 05029, Republic of Korea;; ^e^Department of Materials Science and Engineering, Northwestern University, Evanston, IL 60208;; ^f^Nano Hybrid Technology Research Center, Electrical Materials Research Division, Korea Electrotechnology Research Institute, Changwon 51543, Republic of Korea;; ^g^Accident Tolerant Fuels Technology Development Division, Korea Atomic Energy Research Institute, Daejeon 34057, Republic of Korea;; ^h^Institut Charles Sadron UPR22, CNRS, Université de Strasbourg, F-67000 Strasbourg, France;; ^i^Center for Electronic Materials, Korea Institute of Science and Technology, Seoul 02792, Republic of Korea;; ^j^Materials Sciences Research Center, Japan Atomic Energy Agency, Tokai, Ibaraki 319-1195, Japan;; ^k^Department of Mechanical Engineering, Korea Advanced Institute of Science and Technology, Daejeon 34141, Republic of Korea;; ^l^Research and Development Division, Epicore Biosystems, Inc., Cambridge, MA 02139;; ^m^Department of Chemical and Biomolecular Engineering, University of Illinois at Urbana–Champaign, Urbana, IL 61801;; ^n^School of Mechanical Engineering, Yonsei University, Seoul 03722, Republic of Korea;; ^o^Department of Biomedical Engineering, Northwestern University, Evanston, IL 60208;; ^p^School of Biomedical Engineering, Korea University, Seoul 02841, Republic of Korea;; ^q^Department of Materials Science and Engineering, Querrey Simpson Institute and Feinberg Medical School, Northwestern University, Evanston, IL 60208;; ^r^Department of Biomedical Engineering, Querrey Simpson Institute and Feinberg Medical School, Northwestern University, Evanston, IL 60208;; ^s^Department of Neurological Surgery, Querrey Simpson Institute and Feinberg Medical School, Northwestern University, Evanston, IL 60208;; ^t^Department of Chemistry, Querrey Simpson Institute and Feinberg Medical School, Northwestern University, Evanston, IL 60208;; ^u^Department of Mechanical Engineering, Querrey Simpson Institute and Feinberg Medical School, Northwestern University, Evanston, IL 60208;; ^v^Department of Electrical Engineering and Computer Science, Querrey Simpson Institute and Feinberg Medical School, Northwestern University, Evanston, IL 60208

**Keywords:** healthcare, soft materials, epidermal devices, sweat cortisol, galvanic skin response

## Abstract

Soft microfluidic systems that capture, store, and perform biomarker analysis of microliter volumes of sweat, in situ, as it emerges from the surface of the skin, represent an emerging class of wearable technology with powerful capabilities that complement those of traditional biophysical sensing devices. Recent work establishes applications in the real-time characterization of sweat dynamics and sweat chemistry in the context of sports performance and healthcare diagnostics. This paper presents a collection of advances in biochemical sensors and microfluidic designs that support multimodal operation in the monitoring of physiological signatures directly correlated to physical and mental stresses. These wireless, battery-free, skin-interfaced devices combine lateral flow immunoassays for cortisol, fluorometric assays for glucose and ascorbic acid (vitamin C), and digital tracking of skin galvanic responses. Systematic benchtop evaluations and field studies on human subjects highlight the key features of this platform for the continuous, noninvasive monitoring of biochemical and biophysical correlates of the stress state.

## Significance

Skin-interfaced, wireless devices for clinical-grade monitoring of physiological parameters are of growing interest for uses that range from healthcare to sports performance. This paper introduces a multifunctional skin-mounted microfluidic platform for capture and biomarker analysis of microliter volumes of sweat, a biofluid that can be collected noninvasively, with potential relevance in biophysical sensing. The focus is on colorimetric and digital assessments of a collection of parameters related to stress, including concentrations of vitamin C, cortisol, and glucose, along with quantitative measurements of sweat rate and galvanic skin response. The results represent important additions to a portfolio of emerging capabilities in skin-interfaced technologies for physiological monitoring, with particular relevance to conditions that follow from unhealthy levels of physical and mental stress.

Soft, wearable microfluidic systems with capabilities in colorimetric, fluorometric, and electrochemical sensing of sweat biomarkers offer a range of modalities for tracking performance, nutrition, wellness, and health (1–5). These technologies exploit the rich mixture of solutes, metabolites, hormones of eccrine sweat, and its noninvasive extraction directly from pores on the surface of the skin (6–10). A key requirement for the broad adoption of devices for sweat sensing is in contamination-free capture of precise volumes of sweat and in situ quantitative analysis of multiple biomarkers with relevance to muscle fatigue, dehydration, cystic fibrosis, and others. An important and relatively unexplored frontier focuses on capabilities that support qualitatively expanded domains of application, such as those in tracking biochemical correlates of physical and mental stresses, and other aspects of cognitive status. This area represents the main focus of the results reported here.

Conventional techniques for sweat analysis rely on absorbent pads that adhere to the skin and require subsequent removal, special handling, benchtop centrifugation, and extraction of sweat for off-site analysis (11–13). These laboratory-based strategies are incompatible with real-time monitoring in field settings due to the need for expensive and bulky analysis equipment. Recent developments in advanced, soft forms of microfluidic technologies with integrated chemical and electrochemical sensors serve as the foundations for opportunities in real-time monitoring of various sweat biomarkers and tracking of sweat loss and local rate (3, 14–16). Such devices are thin and flexible, thereby allowing conformal, water-tight coupling to the skin in clinical, athletic, and real-world environments. Related designs that incorporate capillary burst valves and mechanically reinforced stiffening materials (e.g., skeletal designs) enable time sequential analysis (i.e., chronosampling) of multiple sweat biomarkers, and application in demanding scenarios that involve physical impacts (17–19), respectively. In other demonstrations, surface-coated electrodes with antibody- and enzyme-based assays capture information about cortisol and related biochemicals in a continuous mode of operation, although without field studies to demonstrate robustness of operation required for practical applications (20–23).

Simultaneous, reliable analysis of species such as cortisol, together with vitamin C and glucose, has the unique potential to yield insights into transient states of physical and mental stress. Cortisol release from the adrenal glands occurs in response to cognitive and physical stressors. This release activates the sympathetic nervous system (24) and triggers a complex chain of biochemical responses that lead to an increase in energy production (25). In particular, cortisol secretion in response to stress leads to elevated levels of glucose for muscle groups to consume in “fight-or-flight” scenarios. Increased cortisol levels over prolonged time periods, however, have been linked to conditions such as obesity, depression, hypertension, and diabetes (26). Supplementary intake of vitamin C can counteract these harmful effects by boosting the immune response and attenuating cortisol levels (27). A desire to understand the complex relationship between cortisol, glucose, and vitamin C that define dynamic stress responses, motivates the development of devices for noninvasive monitoring of these stress-related biomarkers, as a means for establishing counteractive interventions.

This paper reports technologies that allow measurements of multiple stress-related biomarkers in battery-free, wireless skin-interfaced device platforms. The designs described here include skeletal microfluidic networks with integrated quantitative immunoassays for cortisol and fluorescence assays for glucose and vitamin C, along with features that allow for continuous sensing of sweat rate and conductivity using galvanic skin response (GSR). Demonstrations in benchtop studies and in field trials on human subjects highlight unique capabilities in multimodal and noninvasive monitoring of stress during exercise and at rest in real-world settings.

## Results and Discussion

### Soft, Skin-Interfaced Skeletal Microfluidic Systems with Lateral Flow Immunoassays and Digital Wireless Measurement Capabilities.

**Fig. 1.**
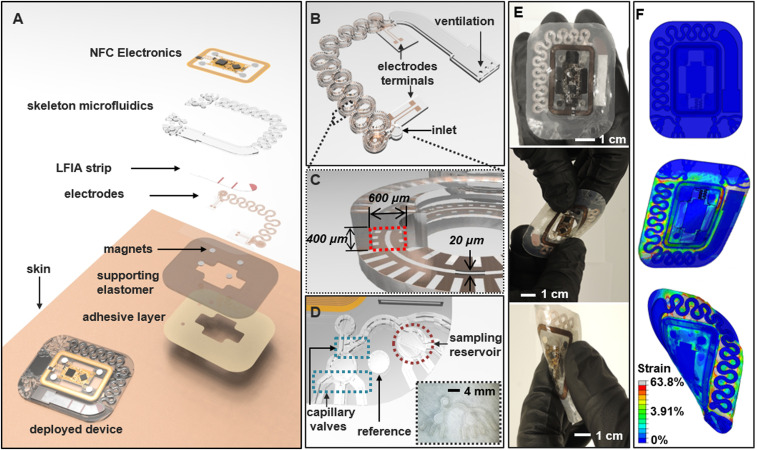
Schematic illustrations and optical images of a skeletal microfluidic device with integrated immunoassays for cortisol, fluorescence assays for glucose and ascorbic acid (vitamin C), and electrical interfaces for sweat loss, sweat rate, and GSR. (*A*) Exploded schematic illustration of the structure of the device. (*B*) Magnified view of the main serpentine skeletal channel for tracking sweat loss, sweat rate, and an immunoassay for cortisol. (*C*) Cross-sectional view of the main channel, highlighting channel dimensions and integrated electrodes. (*D*) Microfluidic structures for fluorescence assays of glucose and ascorbic acid and an optical image of the system (*Inset*). (*E*) Optical image of an assembled device (*Top*), undergoing mechanical twisting (*Middle*) and bending (*Bottom*). (*F*) Three-dimensional modeling of the mechanics associated with similar configurations: flat (undeformed; *Top*), twisted (*Middle*), and bent (*Bottom*) to show the corresponding distributions of strain.

Skin-interfaced systems with integrated immunoassays for sweat cortisol, fluorescent assays for glucose and ascorbic acid (vitamin C), and with electrochemical sensors, support an important range of capabilities for analysis of sweat biomarkers and sweat dynamics related to stress. These multimodal features in sensing exploit ruggedized microfluidic structures formed using a high-modulus (∼1 GPa), ultraviolet (UV) curable polyurethane (NOA81) embedded in a low-modulus (∼30 kPa; Ecoflex; Smooth-On) silicone polymer matrix (28, 29). Fig. 1*A* shows an exploded view of this “skeletal” microfluidic design. Compared to systems based on traditional elastomers, these polyurethane-based microfluidic structures greatly reduce the rate of evaporation of collected and stored sweat (29, 30) (*SI Appendix*, Fig. S1 *A* and *B*) and serve as fluidic connections for embedded lateral flow immunoassays (LFIAs). *SI Appendix*, Fig. S1 *C* and *D* shows a schematic illustration and an optical image of the skeletal microfluidic channels, respectively. The microfluidic channel stores ∼70 µL, and electrodes within these channels establish contact with sweat for continuous, resistive measurements of sweat rate. Additional structures define microreservoirs and capillary burst valves for fluorescence-based measurements of glucose and ascorbic acid. A medical-grade skin adhesive layer with patterned openings attaches the device to the skin and defines access points for collection of sweat directly from skin pores, at positions aligned to inlets on the backside of the device (16). *SI Appendix*, Fig. S2 shows benchtop results of a device filling with artificial sweat at a rate of ∼2 µL/min. An integrated system with electrodes, serpentine microchannels (600-µm width; 400-µm depth), and an LFIA for cortisol measurement appears in Fig. 1 *B* and *C*. Fig. 1*D* illustrates the design of the microfluidic assembly for fluorescence-based glucose and ascorbic acid assays.

Measurements of sweat loss and GSR exploit a collection of ultrathin electrodes (3 µm thick) defined photolithographically using a tacky formulation of polydimethylsiloxane (PDMS) (30:1 mixture of base to curing agent; Sylgard 184; Dow Corning) as a temporary carrier. These patterned electrodes bond to the polyurethane in a UV curing process as shown in *SI Appendix*, Fig. S3*A*. *SI Appendix*, Fig. S3*B* presents images of electrodes after UV curing (top) and of a representative device with integrated electrodes (bottom). *SI Appendix*, Fig. S4 shows results from a simple peel test (Mark-10 ESM1500; ABQ Industrial L.P.; *SI Appendix*, Fig. S4 *A*–*C*) that indicate levels of adhesion between the electrodes and the NOA81 substrate are approximately six times stronger than those associated with bonding to a tacky piece of PDMS (*SI Appendix*, Fig. S4 *D* and *E*). Aligned bonding of the relief side of the prepared structure to the electrode layer yields an enclosed skeletal microfluidic channel system with integrated electrode interfaces (*SI Appendix*, Fig. S3*C*). The electrodes integrate along the serpentine microchannels with direct electrical interfaces to the sweat (Fig. 1*B*) as illustrated in *SI Appendix*, Fig. S5*A*. The system includes reference electrodes (① in *SI Appendix*, Fig. S5*A*), tracking electrodes (④ in *SI Appendix*, Fig. S5*A*), and a counter electrode (② or ③ in *SI Appendix*, Fig. S5*A*). *SI Appendix*, Fig. S5*B* shows magnified optical images of the reference (top) and tracking electrodes (bottom).

The packaging scheme highlighted in *SI Appendix*, Fig. S3*D* and *SI Appendix*, Fig. S6*A* involves steps to embed the microfluidic structure within silicone (Ecoflex; 1:1 mixing, cured at room temperature for 12 h). A laser cutting process defines the perimeter of the resulting system (Fig. 1*E* and *SI Appendix*, Figs. S3*D*, S7*B*, and SI Note 1). Fig. 1*E* shows the device during mechanical twisting and bending. Fig. 1*F* and *SI Appendix*, Fig. S6 *C*–*E* present results of finite-element analysis of the associated mechanics (*SI Appendix*, SI Note 2). The serpentine geometries of the microfluidic channels and the low modulus, stretchable silicone matrix (∼30 kPa) facilitate high levels of elastic deformations (31–33) (*SI Appendix*, Fig. S6 *C*–*E*).

### LFIA for Cortisol.

The cortisol immunoassay relies on a competitive reaction that exploits anti-mouse IgG (anti-IgG) antibody, cortisol-conjugated BSA (cortisol–BSA), and gold nanoparticles (AuNPs) (*SI Appendix*, Fig. S7*A*) with conjugated anti-cortisol antibodies (ACA) (34–36). *SI Appendix*, Fig. S7 *B* and *C* schematically illustrates the reagents and immunoreactions. The ionic affinity of the hydrophobic surfaces of the AuNPs facilitates preparation of ACA–AuNP conjugates via spontaneous reaction of ACA and AuNP at pH ∼7.2, ∼23 °C, and 35% humidity. The immunoassay initiates as ∼90% of collected sweat (∼60 µL) from the main channel reaches the conjugation pad, and cortisol in the sweat conjugates with the ACA–AuNP (cortisol–ACA–AuNP). Sweat then wicks along the nitrocellulose (NC) membrane to launch immunoreactions that occur along control and test lines defined on the membrane. The control line screens uncoupled ACA–AuNP by immobilizing the ACA active sites via cortisol–BSA. The test line senses cortisol–ACA–AuNP quantitatively as a result of binding to anti-IgG (34) (*SI Appendix*, Fig. S7 *B* and *C*). Since there are multiple binding sites per AuNP, the sensitivity and dynamic range of the assay depend on the number of AuNPs, the number of binding sites per AuNP, the total amount of cortisol–BSA, and the concentration of sweat cortisol.

**Fig. 2.**
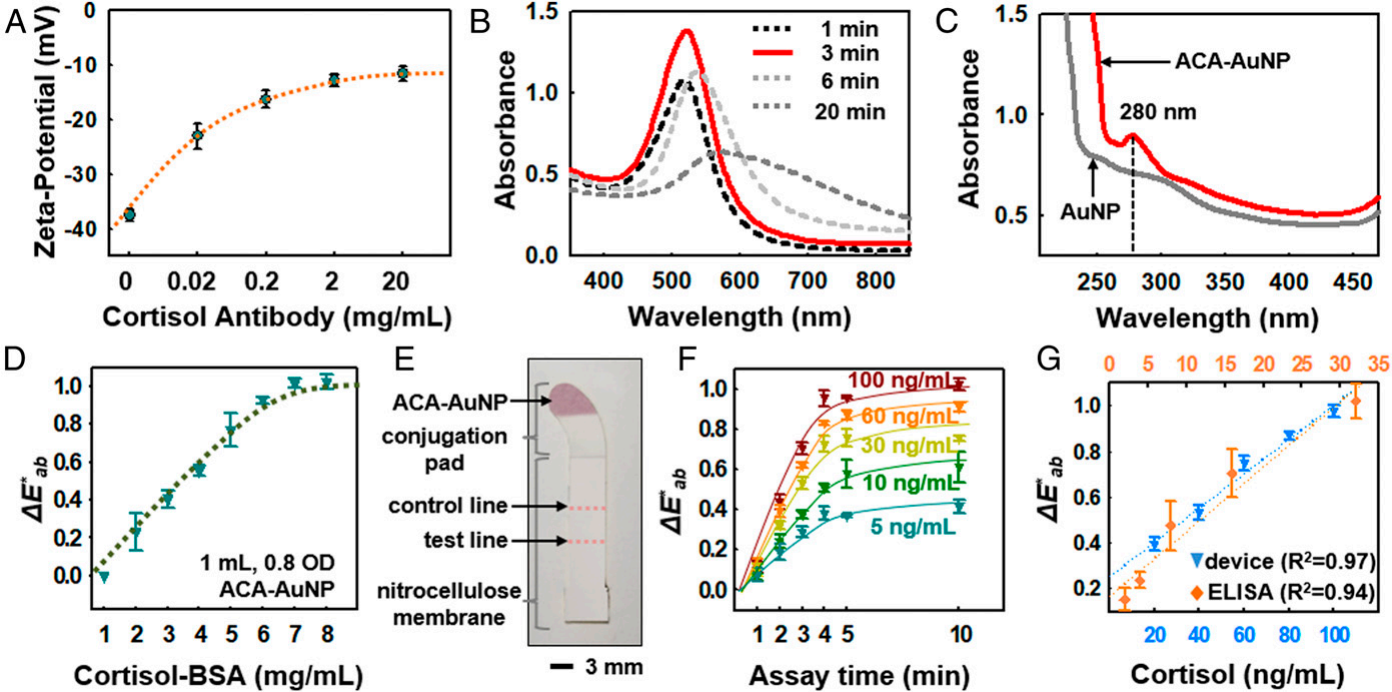
Immunoassay-based lateral flow design and measurements for sweat cortisol. (*A*) ζ-Potential values measured after conjugation of ACA (0, 0.02, 0.2, 2, and 20 mg/mL; three data points for each ACA concentration; *n* = 15) on 30-nm AuNPs. (*B*) Effects of ACA (0.5 mg/mL) conjugation time on absorbance. (*C*) Comparison of absorbance at a wavelength of ∼280 nm before and after ACA conjugation. (*D*) Color development of ACA–AuNP at various concentrations of cortisol–BSA on the NC membrane. (*E*) Optical image of the LFIA strip after assembly and laser cutting. (*F*) Color development trends at various cortisol concentrations (5, 10, 30, 60, and 100 ng/mL) as a function of time. (*G*) Calibration of color index from the device at various concentrations of cortisol (20, 40, 60, 80, and 100 ng/mL) and comparisons to benchtop ELISA tests at concentrations of 2, 4, 8, 16, and 32 ng/mL.

Fig. 2*A* highlights the ζ-potential as a function of ACA concentration (0.02, 0.2, 2, and 20 mg/mL ACA). The error bars are mean values across three samples with SDs for each concentration condition for conjugation of AuNPs (diameters of 30 nm, with highest optical density at ∼550-nm wavelength) as shown in *SI Appendix*, Fig. S7*A*. Increasing the concentration of ACA increases the surface charge, until saturation at ∼2 mg/mL ACA. These measurements indicate that ∼0.5 mg/mL ACA (approximately −20 mV of ζ-potential) is a good choice for conjugation of ACA and AuNPs (37, 38). Fig. 2*B* shows the absorbance spectrum for ACA–AuNP samples for various times of conjugation. The results reveal the time dependence of AuNP aggregation, and the corresponding time required for saturation of the color response (39, 40) (*SI Appendix*, Fig. S8*A*). Insufficient conjugation time produces samples with absorbance lower than those processed in an optimized manner (3 min, as in Fig. 2*B*). Fig. 2*C* shows that the peak absorbance occurs at ∼280 nm, a wavelength range where aromatic groups in the amino acid residues and antibodies absorb strongly (40–42). Transmission electron microscope images (*SI Appendix*, Fig. S8*B*) compare AuNPs before (left) and after conjugation (right), and the image after conjugation (right) shows development of ACA on the AuNP surface. *SI Appendix*, Fig. S8*C* shows the effects of physiologically relevant changes in sweat pH (pH 5.0, 6.0, 7.0, and 8.0) on ζ-potentials for samples of ∼0.5 mg/mL ACA conjugated with 30-nm AuNP. The shift in ζ-potentials is approximately −25 mV to approximately −41 mV for changes in pH from 5.0 to 8.0, which is based on the carboxyl groups and hydroxyl groups being ionized in the shifting alkaline condition (43).

The lateral flow strip consists of a conjugation pad (glass fiber; GFB-R7L; mdi Membrane Technologies; *SI Appendix*, Fig. S9*A*) and a NC membrane (10-µm pore size) with color development at the cortisol–BSA control line (*SI Appendix*, Fig. S9*B*). The absorbent pad confines the reaction system within the NC membrane to ensure rapid and accurate immunoassay reactions. Selection of the membrane material and pore size follow considerations based on the Lucas–Washburn model, according to the following (44, 45):

$$L^{2}\;=\;\gamma rt\cos\theta /2\eta ,$$ [1]

where *L* is the absorption distance, *γ* is the surface tension, *r* is the pore radius, *θ* is the contact angle between the membrane material and the solution, *t* is the time, and *η* is the dynamic viscosity of the solution. Once the collected sweat activates LFIA reaction from conjugation pad, the overall reaction time remains constant, consistent with the Lucas–Washburn model and independent of sweat rate. *SI Appendix*, Fig. S9 *C*–*E* shows the process for assembly of the LFIA strip and optical images before and after laser cutting. A dispensing process delivers cortisol–BSA and anti-IgG to the control and test lines, respectively. The immunoassay design and detection range depend on the amount of immobilized cortisol–BSA on the control line and the active surface areas of both the control and test lines. Color development on the control line changes as a function of cortisol-BSA concentration (Fig. 2*D*). Fig. 2*D* shows the color versus concentration relationship whereby the onset of color saturation for cortisol-BSA occurs at ∼7 mg/mL for a test strip prepared with 1 mL of 0.8 OD ACA–AuNP (*SI Appendix*, Fig. S9*D*; 6 × 1 cm of glass fiber). Accurate colorimetric evaluation of the LFIA involves analysis of images captured with a smartphone, after correcting for ambient lighting conditions (e.g., direct sun light, shade, indoor lighting, transmission properties through the polyurethane microchannel; *SI Appendix*, Eq. **S1**) and with the NC membrane surface as a white reference (*SI Appendix*, SI Note 3) (5, 46).

Fig. 2*E* shows an image of completed LFIA strip and *SI Appendix*, Fig. S10*A* shows the strip integrated in a device for measurements across the physiological range of cortisol concentrations at the test line (Fig. 2*F*; 5, 10, 30, 60, and 100 ng/mL; 50 µg/mL anti-IgG immobilized at the test line). Pictures of the LFIA strips after 10 min of color development appear in *SI Appendix*, Fig. S10*B*. Tests with volunteer subjects indicate the ability to measure cortisol concentrations accurately using this approach, as an alternative to the enzyme-linked immunosorbent assay (ELISA) test, which is a reliable benchtop cortisol assay, as shown in Fig. 2*G* (47).

### Fluorescence-Based Assays for Glucose and Ascorbic Acid.

The device also supports fluorescence-based assays for glucose and ascorbic acid. A pair of reservoirs connected by microchannels and capillary burst valves enable time-sequential sampling of sweat for these measurements. The passive valve geometries have lateral dimensions (∼50 µm) that are significantly smaller than those of the microchannels leading into the reservoirs (150 µm). The burst pressure mechanism follows from the Laplace–Young equation (Eq. **2**) according to the following:

$$Burst\mathrm{Pr}essure\;=\;-2\sigma \left[\frac{\cos\theta_{I}^{*}}{b}+\frac{\cos\theta_{A}}{h}\right],$$ [2]

where σ is the surface tension of liquid, $\theta_{A}$ is the contact angle of the channel, $\theta_{I}^{*}$ is the min [$\theta_{A}$+β; 180°], β is the diverging angle of the channel, and b and h are the width and the height of the diverging section, respectively (30, 48, 49). *SI Appendix*, Fig. S11*A* illustrates the overall design of this network of channels, assays, and the capillary burst valves where “Valve#1” and “Valve#2” have diverging angles of 90° and 120°, respectively. Magnified optical images for the valves are shown in *SI Appendix*, Fig. S11*B*. Tailoring the burst pressures for these valves ensures ordered routing of sweat as it fills into the reservoirs. A separate set of circular reservoirs not connected to the microfluidic network serve as fluorescence reference markers prefilled with fluorescent dye (5 mg/mL tetramethylrhodamine, ethyl ester, perchlorate [TMRE]; Thermo Fisher Scientific) (50).

**Fig. 3.**
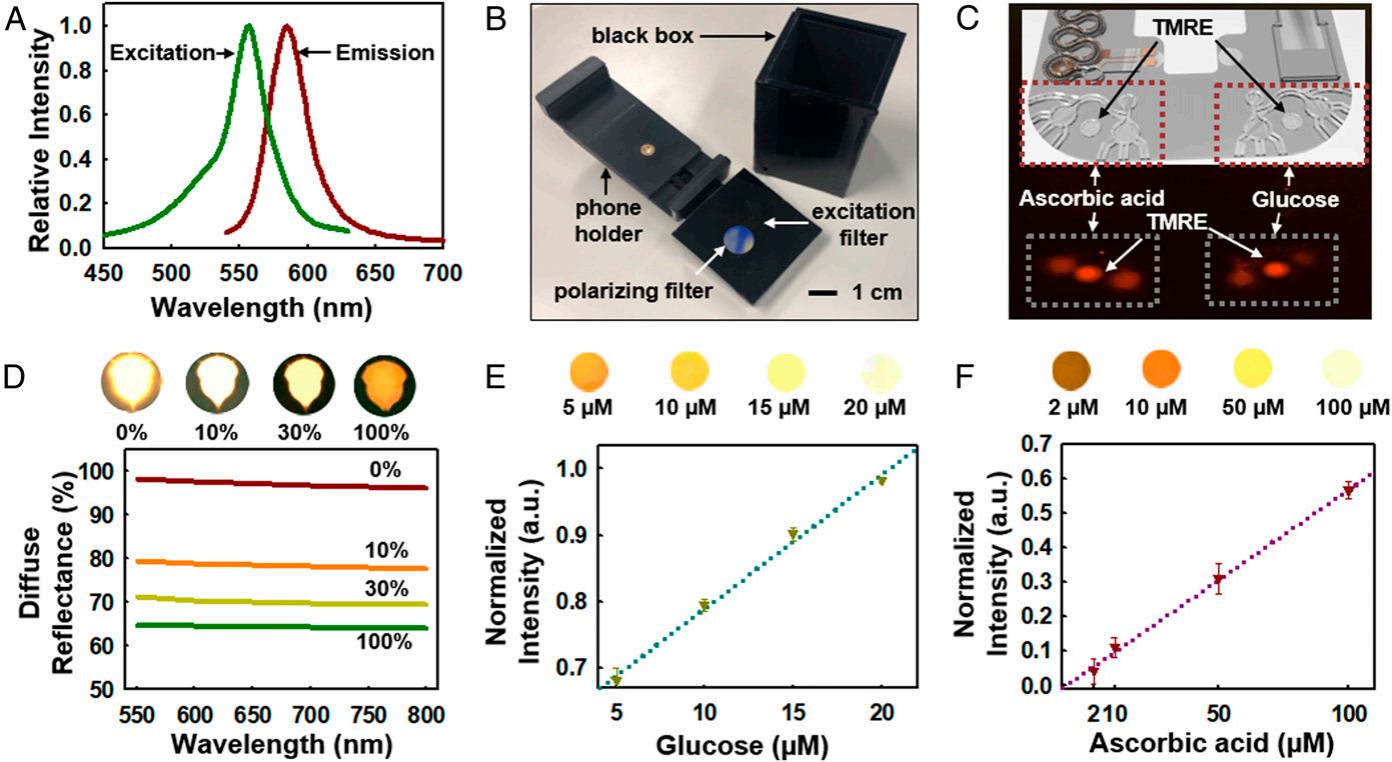
Fluorescence assay design and measurements for sweat glucose and ascorbic acid. (*A*) Excitation and emission curves of OxiRed, the fluorescence probe. (*B*) Optical image of the apparatus used for fluorescence readout. (*C*) Image of ascorbic and glucose signals along with the reference (TMRE) signal. (*D*) Effect of the silicone packaging on the fluorescent signal for various ratios at black and white pigments at 0:10, 1:9, 3:7, and 10:0 (0, 10, 30, and 100%, respectively), along with corresponding images (*Top*). (*E*) Plot of the normalized fluorescence intensity for various glucose concentrations at 0.1, 0.5, 1, and 2 µM and their fluorescence intensities from associated images (*Top*). (*F*) Plotting of normalized fluorescence intensity for various ascorbic acid concentrations at 5, 10, 50, and 100 µM concentrations and their fluorescence intensities from associated images (*Top*).

*SI Appendix*, Fig. S12 *A* and *B* summarizes reactions that involve glucose and ascorbic acid with glucose oxidase (GOx) and ascorbic acid oxidase (AOx) enzymes, respectively. The oxidation reactions for both substrates generate hydrogen peroxide, and excess activity of horseradish peroxidase (HRP) leads to reduction of a fluorometric probe (OxiRed) to form resorufin as the basis of a fluorescence signal (Fig. 3*A*; *λ*_excitation_, ∼550-nm wavelength, and *λ*_emission_, ∼600-nm wavelength) with magnitude that depends on the concentration (51) (*SI Appendix*, Fig. S12*C*). *SI Appendix*, SI Note 4 summarizes the details of enzymatic preparations for glucose and ascorbic acid assays. *SI Appendix*, Fig. S12 *D* and *E* shows the effect of pH on the activity of GOx and AOx, respectively. An apparatus with integrated excitation and emission filters attaches to a smartphone to facilitate rapid measurement of the fluorescence in situ (49) (Fig. 3*B*). The emission filter passes only light with wavelengths longer than 610 nm. *SI Appendix*, Fig. S12*F* shows the key features of this module and its coupling to a smartphone imager. This setup facilitates capture of fluorescence signals as measures of the concentration of target substrates (i.e., glucose and ascorbic acid). Fig. 3*C* features signals from glucose and ascorbic acid relative to those from the TMRE reference reservoir. Calibration involves first analyzing the signal intensity from each reservoir and then normalizing these results by the intensity of TMRE (49, 52) (*SI Appendix*, Eq. **S2**). The depth of each microreservoir controls the dynamic range of the fluorescence signal, according to the Beer–Lambert law (53). Fig. 3*D* shows the effect of the silicone packaging on the fluorescent signal at 0:10, 1:9, 3:7, and 10:0 ratios (0%, 10%, 30%, and 100%, respectively) of black and white pigments mixed in uncured Ecoflex at ∼5% (wt/wt). Fig. 3 *E* and *F* shows representative examples of the fluorescence intensity increasing for glucose and ascorbic acid assays in a linear manner over physiologically relevant range of concentrations.

### Battery-Free, Wireless Electronic Interface for Readout of Sweat Rate and GSR.

**Fig. 4.**
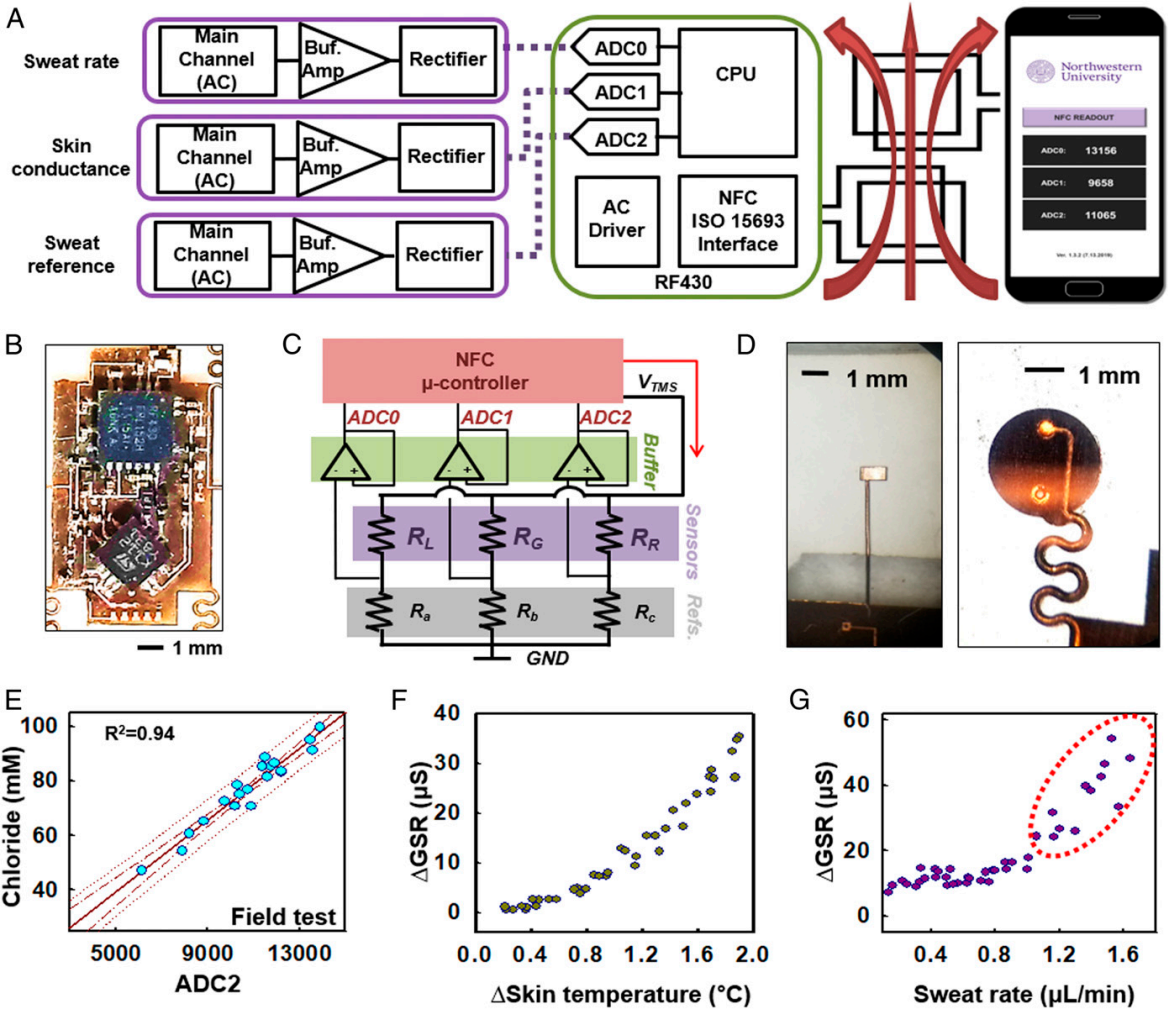
Design of NFC electronics for monitoring sweat loss, sweat rate, and GSR. (*A*) Schematic block diagram of the NFC electronic system and its interface to a sweat microfluidic device and a smartphone. (*B*) Optical image of the electronics to show chip placement. (*C*) Schematic block diagram of the electronics to show the reference resistor layouts for the main, reference, and GSR readout. (*D*) Magnified optical images of the electrode terminals for GSR (*Left*) and tracking reference electrodes that couple with the microfluidic device (*Right*). (*E*) Plot of electrolyte concentration for a series of samples of human sweat in the reference microchannel and corresponding ADC2 values determined by wireless readout. (*F*) Effect of body temperature at the initial phase of exercise on ∆GSR. (*G*) Correlation between sweat rate and ∆GSR after skin temperature stabilizes and sweating begins (forearm, 18 to 20 °C temperature, and 15 to 30% humidity).

Fig. 4*A* highlights the wireless electronic module, which consists of three electrodes (sweat rate, skin conductance, and sweat reference), a near-field communication (NFC) microcontroller (RF430, ISO/IEC 15693, ISO/IEC 18000-3; Texas Instruments), and an RF antenna. The microcontroller receives power wirelessly (*SI Appendix*, Fig. S13 *A*–*D*) from an NFC-enabled device such as smartphone (16, 54, 55). The electrodes deliver alternating current (AC) to the skin to measure GSR, with a common node of *V*_TMS_ for applying an AC driving signal (Fig. 4 *B* and *C*; *R*_*a*_, *R*_*b*_, and *R*_*c*_ for 100, 300, and 10 kΩ, respectively). The digital readout system compares measured resistances to known reference resistors, *R*_*a*_, *R*_*b*_, and *R*_*c*_, to allow for evaluating sweat rate, GSR, and sweat conductivity, respectively (Fig. 4*C*). The analog-to-digital converter (ADC) ports on the NFC microcontroller (RF430, ISO15693 interface) acquire data from the three electrodes of *R*_*L*_, *R*_*G*_, and *R*_*R*_ (Fig. 4*C*). The ADC output voltages for each channel can be described by the following equations:

$$\text{ADC}0=V_{TMS}\times \frac{R_{a}}{R_{a}+R_{L}}-V_{f},\;\;\text{ADC}1=V_{TMS}\times \frac{R_{b}}{R_{b}+R_{G}}-V_{f},\;\;\text{ADC}2=V_{TMS}\times \frac{R_{c}}{R_{c}+R_{R}}-V_{f},$$ [3]

where *R*_*L*_ is the resistance across the pair of electrodes in main channel, *R*_*G*_ is the resistance across the electrodes for GSR, *R*_*R*_ is the resistance at the reference electrode, and *V*_*f*_ is the forward voltage of the rectifier (∼150 mV) (16). Fig. 4*D* shows the terminals for GSR (left; *SI Appendix*, Fig. S13*E*) and the tracking and reference electrodes that couple with the embedded electrodes in the microfluidic channel (right). The acquired data passes wirelessly to the smartphone. *SI Appendix*, Fig. S13*F* provides details on the individual terminals of the RF430 and TSV632 and the layer of PDMS that prevents ingress of external moisture, respectively.

*SI Appendix*, Fig. S14*A* summarizes conductance measurements from ADC0 for artificial sweat collected in the main microchannel for various electrolyte concentrations. The captured sweat volume depends linearly on sweat filling length along the channel (*SI Appendix*, Fig. S6*A*) and, as a result, on the resistance at the tracking electrodes according to the following:

$$L=\alpha \cdot R_{R}/R_{L},$$ [4]

where *L* is the filling length (*L* = 0 to 165 mm) and *α* is a coefficient that accounts for the ratio of the lengths of the reference and tracking electrodes (reference electrode, 1.5 mm; tracking electrodes, 165 mm). Measurements at 1 kHz minimize the dependence of impedance phase on the conductivity of the skin and sweat (16) (*SI Appendix*, Fig. S14 *B*–*D*). Benchtop and field testing with volunteer subjects determine the relationship between ADC2 and sweat conductivity (*SI Appendix*, Fig. S14*E* and Fig. 4*E* for benchtop and field testing, respectively).

Evaluations of skin conductivity using the GSR electrodes, as shown in *SI Appendix*, Fig. S13*E*, and comparison with sweat rate provide important insights into sweat gland activity, including sweat rate and ion reabsorption. Coupling of the electronics module with the skeletal microfluidics enables electronic data collection of sweat rate. The magnets (3-mm diameter, ∼0.5-mm thickness), attached to the electronics module, offer robust magnetic forces for mechanical coupling with the electrode terminals (16) (Fig. 1*A* and *SI Appendix*, Fig. S14*F*). Previous studies demonstrate that sweat electrolyte concentrations increase with increasing sweat rate (55–57). Prolonged exercise on a stationary bike induces high sweat rates, which could give rise to reduced ion reabsorption. Fig. 4*F* shows results that correlate ∆GSR with skin temperature for the initial phases of exercise (∼10 min at 18 °C room temperature). A warmup period of 10 to 15 min leads to sweating and development of a stable skin temperature (58). Fig. 4*G* shows representative ∆GSR data collected from the forearm of a subject. The eccrine glands selectively reabsorb ions, especially sodium, during sweating as the basis of physiological regulation for homeostasis. The constant ∆GSR trend shown in Fig. 4*G* reflects this type of physiological regulation at low sweat rate under ∼0.8 μL/min. Further exercise without hydration induces overperspiration and perturbs the regulation system. The ∆GSR data are consistent with this behavior, as it increases when the sweat rate reaches 0.7 µL/min and the ion resorption rate exceeds the excretion rate (59–62) (Fig. 4*G*).

### Demonstrations and Field Testing.

**Fig. 5.**
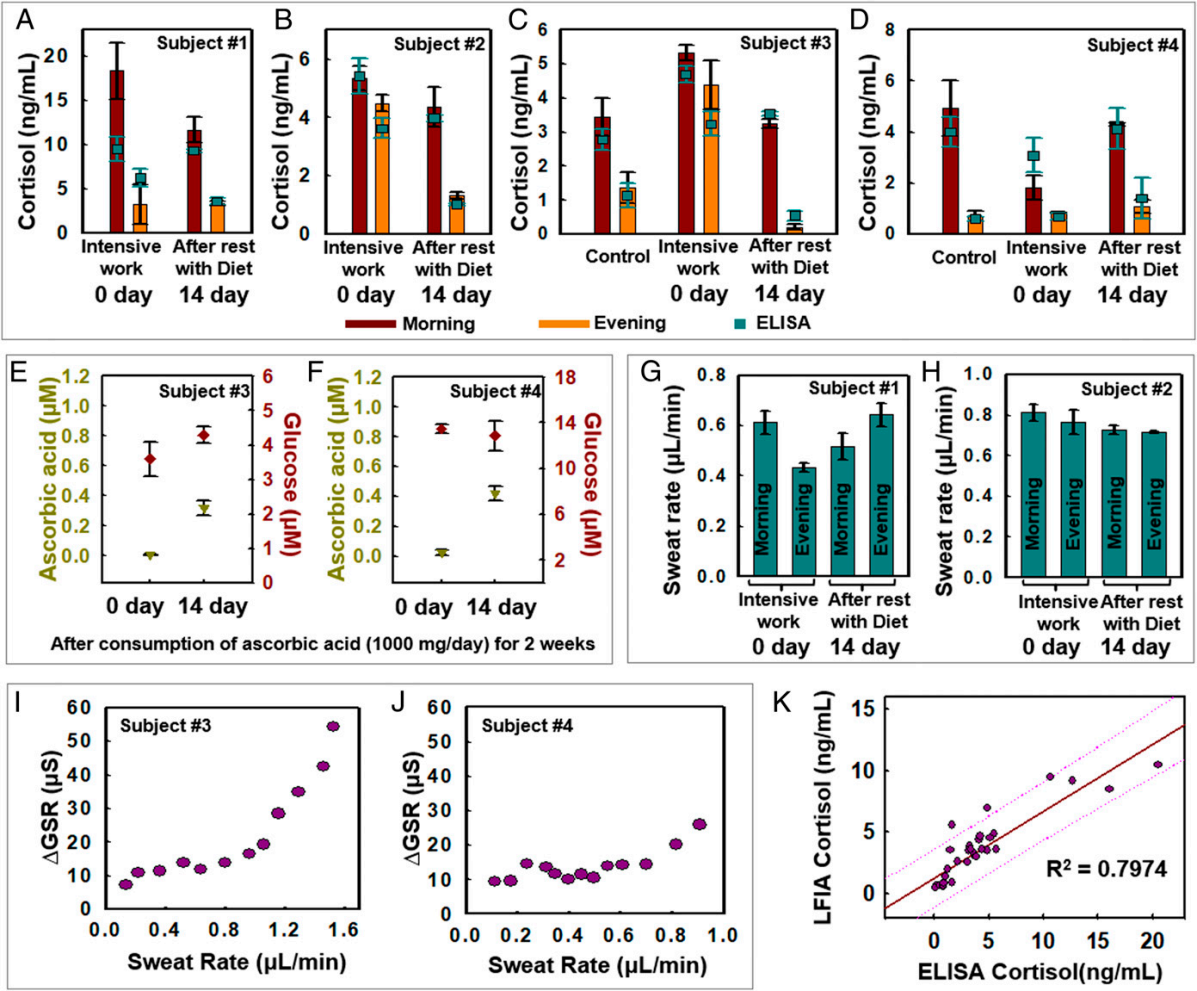
On-body measurements of sweat biomarkers during exercise. (*A*–*D*) Cortisol LFIA results for subjects 1 and 4 at 0 and 14 d. “Control” (*C* and *D*) indicates measurements of sweat cortisol under normal conditions of the subjects not being stressed. (*E* and *F*) Results of ascorbic acid and glucose at 0 and 14 d for subjects 3 and 4. (*G* and *H*) Sweat rate measurements for subjects 1 and 2 at 0 and 14 d. (*I* and *J*) ∆GSR measurements during high-intensity exercise and sweating for subjects 3 and 4. (*K*) Plotting and regression of quantitative assays results from LFIA and ELISA. Dotted line is prediction line.

Field tests illustrate capabilities in measurement of cortisol, glucose, and ascorbic acid along with digital tracking of sweat rate and GSR across four healthy volunteers engaged in physical exercise on a stationary bike in a gym environment (*SI Appendix*, Fig. S15 *A*–*C*; 18 to 22 °C temperature and 15 to 30% humidity). The initial set of experiments involve data collection from subjects 1 and 2 in the morning and evening. Sweating was induced within 30 min after the subject woke up at ∼7 AM and before going to sleep around 7 PM (Fig. 5 *A* and *B*). Intensive work periods ensued for ∼7 d (including overnight work and consumption of caffeine) and rest (regular patterns of sleeping and eating meals) for 14 d, which served as short-term stressors. Additional short-term studies conducted with subjects 3 and 4 focused on cortisol, glucose, and ascorbic acid measurements along with characterization of sweat rate and GSR (Fig. 5 *C*–*J*) in the morning and evening during intensive work, rest with healthy diet, along with a control measurement (using saliva) (63–65).

*SI Appendix*, Fig. S16 shows these sequences of events and methods for capturing sweat information at each stage using a smartphone. The filling of sweat into the microchannels activates the glucose and ascorbic acid assays within ∼10 to 15 min after the start of the stationary bike exercise. Upon filling of the reservoirs for glucose and ascorbic acid assays, a smartphone camera with a shielding module (Fig. 3*B* and *SI Appendix*, Fig. S12*F*) captures the fluorescent signals. As sweat fills the main channel, measurements of sweat rate and GSR (4 to ∼13 separate times) can be performed by placing the smartphone in proximity to the device. The LFIA assay activates after complete filling of the main channel (∼70 µL). The digital camera on the smartphone then captures the developed color.

Fig. 5 *A*–*D* compares measurements of cortisol conducted with the LFIA and a benchtop ELISA protocol in the morning and evening, under intensive work and resting conditions. Under ordinary, routine circumstances, diurnal variations of cortisol level among the subjects exhibit previously observed patterns, whereby concentrations peak in the morning, to facilitate focus during the daytime, and then gradually decrease through the afternoon and evening. This cortisol circadian rhythm is apparent in data from subjects 3 and 4, as shown in Fig. 5 *C* and *D* (64, 65). The subjects experience physiological stress, fatigue, and irregular life patterns during intensive work and with inadequate sleep. These conditions disrupt the cortisol–melatonin circadian rhythm. As a result, the hypothalamus region of the brain produces corticotropin-releasing hormone, which in turn activates the hypothalamic–pituitary–adrenal axis and stimulates anterior pituitary activity (23, 24, 66), thereby increasing cortisol production and disrupting of cortisol circadian rhythm (67). The cortisol levels for subjects 2, 3, and 4 during intensive work show disruptions of circadian rhythm, consistent with physiological conditions that reflect exhaustion (Fig. 5 *B* and *C*). This disruption could aggravate the psychological state (e.g., anxiety, insomnia, etc.). Fig. 5*K* demonstrates the accuracy and reliability of the LFIA in the device compared with benchtop ELISA results (*R*^2^ = 0.7974). The control tests measuring saliva cortisol levels before and after exercise show that the effect of exercise intensity on cortisol level is small (68) compared to circadian rhythm changes that occur during the day. After intense work condition, the subjects return to a routine life pattern for 2 wk and consume ascorbic acid (1,000 mg/d for subjects 3 and 4) (69). The result is that the cortisol patterns recover to normal circadian rhythm, as shown in Fig. 5 *A*–*C*. Although subjects 1 to 3 show lower cortisol levels after 14 d, the relative changes in sweat cortisol levels due to circadian rhythm appear to dominate compared to dietary interventions for subject 4 (Fig. 5*D*). Fig. 5 *E* and *F* shows that the ascorbic acid levels increase from ∼0 to ∼0.33 µM for subject 3 and from ∼0 to ∼0.42 µM for subject 4, as a result of vitamin C intake. By contrast, the glucose levels exhibit no specific trends, i.e., ∼0.62 and ∼0.80 µM for subject 3 and 0.84 and 0.81 µM for subject 4, as mean values of measurements taken on days 0 and 14. *SI Appendix*, Fig. S15 *D* and *E* shows glucose and ascorbic acid measurements at days 0, 2, 6, 10, and 14 for these same two subjects. These results show that the device along with integrated assays have practical utility, as the glucose and ascorbic acid sensitivity ranges are within previously reported physiological ranges (3, 70).

Representative results for sweat rate and ∆GSR appear in Fig. 5 *I* and *J*. These findings establish correlations between ∆GSR and sweat rate, likely associated with resorption and secretion of ions due to sweating. Sweat rate measurements from subjects 1 and 2 appear in Fig. 5 *H* and *I*, with comparisons to ∆GSR in *SI Appendix*, Fig. S15 *F* and *G*. Wirelessly acquired data from ADC0 and ADC2 yield the sweat rate and electrolyte concentrations, respectively. Comparisons of ∆GSR and sweat rate in Fig. 5 *I* and *J* suggest that resorption behavior occurs for secreted ions (i.e., sodium) from the proximal secretory coil (18, 59, 70–72). In the limiting case, the rate of secretion of ions exceeds the rate of resorption, thereby leading to an increase in ∆GSR at a critical sweat rate (73, 74). Fig. 5 *I* and *J* shows that ∆GSR measurements for subjects 3 and 4 remain steady until the sweat rate reaches ∼1.1 µL/min for subject 3 and ∼0.8 µL/min for subject 4, at which point the ∆GSR increases.

## Conclusion

Eccrine sweat is an interesting, yet incompletely understood, class of biofluid that contains a range of chemical species whose concentrations could provide significant information about physiological status. The potential relevance spans sports science, clinical medicine, and military readiness. The multifunctional device platform described here exploits a soft microfluidic network of hard channels and reservoirs, with integrated flexible electronic systems, as a practical laboratory-on-a-chip–type system with immunoassays, fluorometric detection capabilities, and wireless functionality tailored specifically for monitoring physical and mental stresses. A key feature is lateral flow integration for immunoassay analysis of sweat cortisol. The fluorescence assays provide information on other trace chemicals such as glucose and ascorbic acid. Wireless modes of operation based on NFC protocols also support real-time digital tracking of sweat rate and GSR. Field tests on human subjects engaged in activities to induce and then relieve stresses demonstrate the utility of the technology in this important context, as well as its ability to address scenarios of practical interest. The versatile multimodal design principles introduced here can be configured to address many additional capabilities in sweat collection, storage, and chemical analysis in remote field settings (e.g., modified skin mounted microfluidics, in which microphotodetectors and excitation light sources are embedded to enable fluorescent readouts).

## Materials and Methods

### Fabrication of Soft Skeletal Microfluidics with Flexible Electrodes System.

Fabrication began with the formation of a mold from a silicon wafer patterned using photolithography techniques. More precisely, photoresist KMPR1010 was spin-cast on a silicon wafer at 3,000 rpm for 30 s, baked on a hot plate at 110 °C for 3 min, exposed to UV irradiance at 300 mJ⋅cm^−2^ for 2 min, and developed with developer MF917. Deep reactive-ion etching (polymethylmethacrylate coating; STS Pegasus ICP-DRIE; SPTS Technologies Ltd.) removed the exposed silicon to a selected depth (∼400 µm). A prepolymer to PDMS (Sylgard 184; Dow Corning; mixed at a 10:1 ratio of base to curing agent by weight) was then cast and cured on the silicon structures to yield soft molds. These molds were used to form structures of NOA (Norland Optical Adhesive; NOA 81; Norland Products; partial curing; expose to 30-W UV light for 4 to ∼10 min; *SI Appendix*, Fig. S1*B*).

Fabrication of Cu electrodes relied on a photolithographic process, whereby photoresist (AZ4620) was spin-cast at 3,000 rpm for 30 s, and then baked on a hot plate at 60 °C for 1 min. After UV irradiance at 300 mJ⋅cm^−2^ to expose the photoresist, a development process for 1 min yielded the desired pattern. Next, wet etching with copper etchant (HFCE100; Transense) of Cu foils (DuPont) laminated onto glass slides (Fisherbrand) coated with PDMS (Sylgard 184; Dow Corning; mixed at a 20:1 ratio of base to curing agent by weight and partially cured on a hot plate at 110 °C for 3 min; *SI Appendix*, Fig. S3*A*) removed the exposed regions of the Cu. Casting a ∼500-µm-thick layer of NOA 81 on the patterned Cu–PDMS substrate and exposing to UV light (30 W for 4 min) enabled transfer of the Cu electrodes to the surface of the NOA 81 (*SI Appendix*, Fig. S3*A*). Assembly of NOA microfluidic trenches (*SI Appendix*, Fig. S1*D*) and electrodes (*SI Appendix*, Fig. S3*B*) exploited uncured NOA81 to create sealed channels with precise alignment (*SI Appendix*, Fig. S3*C*). A laser cutter (ProtoLaser R; LPKR) defined the perimeter of the assembly as the final step to complete the fabrication (*SI Appendix*, SI Note 4).

### LFIA Platform Preparation.

The addition of 1 M NaOH to colloidal 30-nm gold nanoparticles (GNPs) (Sigma-Aldrich) shifted the pH close to 7.0. Adding 0.5 mg/mL anti-cortisol antibody (ACA) (ab1951; Abcam) to a final concentration of ∼0.5 µg/mL and incubating (rotating at 30 rpm) the solution for 1, 3, and 20 min enabled spontaneous conjugation of antibody onto the activated GNP. Adding 10% (wt/vol) BSA (to final concentration of 0.1%; Sigma), allowing stabilization at room temperature for 1 h, centrifuging (9,000 × *g* for 30 min at 4 °C, followed by approximately four times repetition of washing–resuspending of precipitated pellet with a storage buffer; PBS buffer includes 1% BSA and 2% sucrose), and drying of the separated precipitation at room temperature for 4 h yielded anti-cortisol antibody-conjugated gold nanoparticles (ACA–GNPs) (stored at 4 °C).

Cortisol-BSA and IgG antibody were immobilized on a nitrocellulose membrane (pore size: 5, 8, 10, 12, and 15 µm; Advanced Microdevices) as the control and test lines, respectively (Claremont BioSolutions). A sample conjugation pad (Advanced Microdevices) was saturated with ACA–GNPs for 1 h and then dried for 30 min at 37 °C. The prepared nitrocellulose membrane and an absorbent pad (filter paper no. 1; Whatman; GE Healthcare Life Sciences), as shown in *SI Appendix*, Fig. S9*C*, served as supports for the LFIA. Standard protocols for ACA, cortisol–BSA, IgG antibody, and BSA set a 1-y shelf life from when the package is delivered.

### Electronics Design and Assembly.

Fabrication began with patterning of a two-layer printed circuit board by processing of multilayer foils of Cu–PI–Cu (18 μm/75 μm/18 μm) with a UV laser cutter (ProtoLaser U4; LPKF). The main processor, RF430FRL152HCRGER (RF430, ISO/IEC 15693, ISO/IEC 18000-3; Texas Instruments), served as the NFC platform, with the ability to rectify incident power from a smartphone device at up to 720 µW at 2.0 V, depending on coupling efficiency, and relaying data over the 13.56-MHz communications link. The RF430 supports 14-bit Sigma-Delta ADC with triple analog inputs at an input range up to 900 mV and maximum sampling frequency of 2 kHz, down-sampled to 1-Hz resolution. Signal amplification and measurement of the main and reference channels used another chip, TSV634QFN16 (STMicroelectronics), as a four-channel operation amplifier with low energy consumption (60 μA at 5 V) and large unity gain-bandwidth (800 kHz). On power-up, the system sourced a 5-kHz, rail-to-rail square wave to the channels, causing an AC current to flow through the collected sweat. The magnitude of this current is proportional to the concentration of ions in sweat, as an electrical impedance that causes the sourced 5-kHz waveform to attenuate during passage through the sweat. This attenuation reduces the amplitude of the waveform whose rectified voltage is buffered and measured by the TSV634 and ADC, respectively. This rectified voltage level is sampled, processed, and relayed to an NFC compatible reader by the RF430. An NFC-compatible smartphone (LG Nexus 5X; LG) and custom-developed application for the Android operating system enabled visualization of the data.

### Field Studies.

Field studies were conducted on four healthy volunteers exercising on stationary bikes in a gym environment (18 to 20 °C temperature and 15 to 30% humidity). To characterize the effects of mental stress (i.e., long studying or research time with irregular sleeping pattern), subjects consumed ascorbic acid supplements (1,000 mg/d) for 14 d (75). All subjects provided signed consent and had medical consultations before and after field tests with a medical doctor. This study was approved by the Institutional Review Board (IRB: STU00208494) at Northwestern University. Control tests using saliva samples extracted from the subjects were used to verify circadian rhythm changes observed in sweat cortisol levels. Prior to mounting the devices, the skin was cleaned with 70% ethanol. Subjects wore sportswear (shorts and T-shirt) for the tests.

## Supplementary Material

Supplementary File

## Data Availability

All study data are included in the article and *SI Appendix*.
