# Supplementary material for: Soft, skin-interfaced microfluidic systems with integrated immunoassays, fluorometric sensors, and impedance measurement capabilities
Source: Proc Natl Acad Sci U S A. 2020 Oct 26;117(45):27906–15. doi: 10.1073/pnas.2012700117 (PMC7668081; doi:10.1073/pnas.2012700117)
Supplement: Supplementary File [file pnas.2012700117.sapp.pdf]

PNAS
www.pnas.org

Supplementary Information for

Soft, skin-interfaced microfluidic systems with integrated immunoassays, fluorometric sensors and impedance measurement capabilities

Sungbong Kim[a,b,1], Boram Lee[c,1], Jonathan T. Reeder[a,d,1], Seon Hee Seo[e,1], Sung-Uk Lee[f], Aurélie Hourlier-Fargette[a,d,g], Joonchul Shin[h], Yurina Sekine[i], Hyoyoung Jeong[a,d], Yong Suk Oh[a,j], Alexander J. Aranyosi[a,k], Stephen P. Lee[a,k], Jeffrey B. Model[a,k], Geumbee Lee[a,d], Min-Ho Seo[a,d], Sung Soo Kwak[a,d], Seongbin Jo[l], Gyungmin Park[l], Sunghyun Han[l], Inkyu Park[j], Hyo-Il Jung[m], Roozbeh Ghaffari[a,k,n,*], Jahyun Koo[o,*], Paul V. Braun[b,*], John A. Rogers[a,k,p,*]

[1] S.K., B.L., J.T.R, and S.H.S. contributed equally to this work.
[*] To whom correspondence may be addressed. E-mail: rooz@northwestern.edu, jahyunkoo@korea.ac.kr, pbraun@illinois.edu, jrogers@northwestern.edu

This PDF file includes:

Supplementary text
SI Appendix Figures S1 to S16

www.pnas.org/cgi/doi/10.1073/pnas.2012700117

Supplementary Text (SI Note)

SI Note 1. Device Assembly and Packaging. Encapsulating the NOA microfluidic system within a low modulus silicone polymer (*SI Appendix*, Fig. S3*D*) yielded a soft platform with elastic mechanical properties. An overcoat of PDMS created a waterproof covering. A double-sided skin adhesive (PC2723U; ScapaHealthcare, CT) with circular openings defined by a CO_2 laser cutter (Universal Laser Systems, AZ) and aligned with the inlets on the bottom side of the device served as a skin interface. The adhesive attachment to the device was enhanced by exposure to O_2 plasma (Harrick Plasma Oxygen Cleaner, Ithaca, NY) using 18 W RF power. When stored with silica gel at low humidity (<5%) and temperature (4°C), the devices maintain performance for ~6 months.

SI Note 2. Finite element analysis (FEA) and mechanics modeling of the microfluidic structures. ABAQUS commercial software (Dassault Systems) was used to perform 3D finite element analysis (FEA) and mechanics modeling of the devices under different external loads (bending and twisting). The displacement field applied to the bottom surface of the skin corresponded to 2-cm bending radius of curvature, and each end of the device was subjected to 90-degree rotation such that the two ends of the skin had a 180-degree rotation for twisting. Eight-node 3D solid elements were used for the skin and the microfluidic system, while four-node shell elements were used for the electronics. Young's modulus and Poisson's ratio used in the simulations were 29.5 kPA and 0.4999 for EcoFlex, 420 kPa and 0.5 for PDMS, 1 GPa and 0.43 for NOA81, and 4 GPa and 0.34 for Cu-PI circuit board, respectively.

SI Note 3. Analytical methods. Bench-top analysis of glucose and ascorbic acid concentrations relied on liquid chromatography-mass spectrometry systems (LC-MS; Waters Synapt G2-Si ESI, MA). An Aminex HPX-87H ion exclusion column (Bio-Rad, Hercules, CA) and a refractive index detector enabled evaluations of glucose at a 0.8 mL/min flow rate of 0.005 N H_2SO_4 eluent and a 50 °C column temperature. The total sample volume was 100 μL with at least 10 μL of sweat. An ACE C18 analytical column of 150×4.6 mm and 3 m particle size, 300 ̊A with 15 μL injection volume at 20 °C at 0.4 mL/min flow rate of an eluent; 3:7 ratio of methanol and water) served as a separation column for ascorbic acid with a UV detector at λ=251 nm. Control quantitative assays of sweat cortisol relied on commercial ELISA kits (Cortisol competitive Human ELISA kit, Invitrogen, Carlsbad, CA) with ~ 1 mL sweat samples collected with absorption pad, separately. A halide analyzer enabled measurement of chloride concentration, using an ion-specific electrode (Orion, Thermo Scientific, Walthan, MA) calibrated to the ppm level.

Quantitative analysis of cortisol concentration relied on RGB values extracted from digital images of the lateral flow immunoassay strip using commercial software (Photoshop, Adobe, San Jose, CA). The white nitrocellulose membrane of the lateral flow assay served as a white color reference, and yielded L*a*b* values according to the following equation:

$$\Delta E_{ab}^{*} = \sqrt{(L_n^* - L_0^*)^2 + (a_n^* - a_0^*)^2 + (b_n^* - b_0^*)^2} \quad \text{(Eq. S1)}$$

where L^*_n, a^*_n, and b^*_n denote the values at the nth assay location and L^*_0, a^*_0, and b^*_0 are values for the white reference region. Sampling of 5-6 indexes of RGB values from the captured image (from the darkest to the brightest points in the histogram) used commercial image software tools (Adobe Photoshop®). This technique enabled derivation of mean values with standard deviations as the basis of the colorimetric analysis data.

Quantitative analysis of glucose and ascorbic acid concentrations relied on image analysis software (Image J, NIH, USA) that correlates the fluorescent signal intensity to the concentration of each analyte according to the following equation:

$$Normalized\ FL\ intensity\ = I_{sample}\ /\ I_{reference} \quad \text{(Eq. S2)}$$

where I_{sample} and $I_{reference}$ denote the measured intensities from the microchambers of sample and reference, respectively.

Here, color analyses of liquid samples were conducted with a Varian Cary5G UV–Vis–NIR spectrophotometer (Agilent, Santa Clara, CA).

SI Note 4. Preparation of assays for glucose and ascorbic acid. A mixture of ascorbate oxidase from *Cucurbita sp.* (AOx; 20 mg/mL; solubilized in 0.1 M sodium phosphate buffer; pH 5.6; Sigma-Aldrich, St. Louis, MO) and horse radish peroxidase (HRP; 30 mg/mL; BioVision Inc., Milpitas, CA) defined an enzyme cocktail for the ascorbic acid assay. An enzyme cocktail for the glucose assay included glucose oxidase from *Aspergillus niger* (GOx; 5 mg/mL; solubilized in 0.1 M sodium phosphate buffer; pH 6.0; Sigma-Aldrich, St. Louis, MO) and HRP (30 mg/mL). Small volumes of the optimized dose of the enzyme mixtures (~ 1 µL) and OxiRed™ (~ 1 µL; BioVision Inc., Milpitas, CA), which served as the fluorescence probe, were separately placed into each reservoir and then dried in a desiccator at room temperature for ~4 h. Tetramethylrhodamine, ethyl ester (TMRE; 1 mM; Thermo Fisher Scientific, Waltham, MA) was loaded into the reference chamber by drop casting (~0.5 µL), as a reference fluorescent reagent. The enzymes and fluorescent probe are viable for use for up to 1 year after purchase.

Supplementary Information Appendix Figures (SI Appendix)

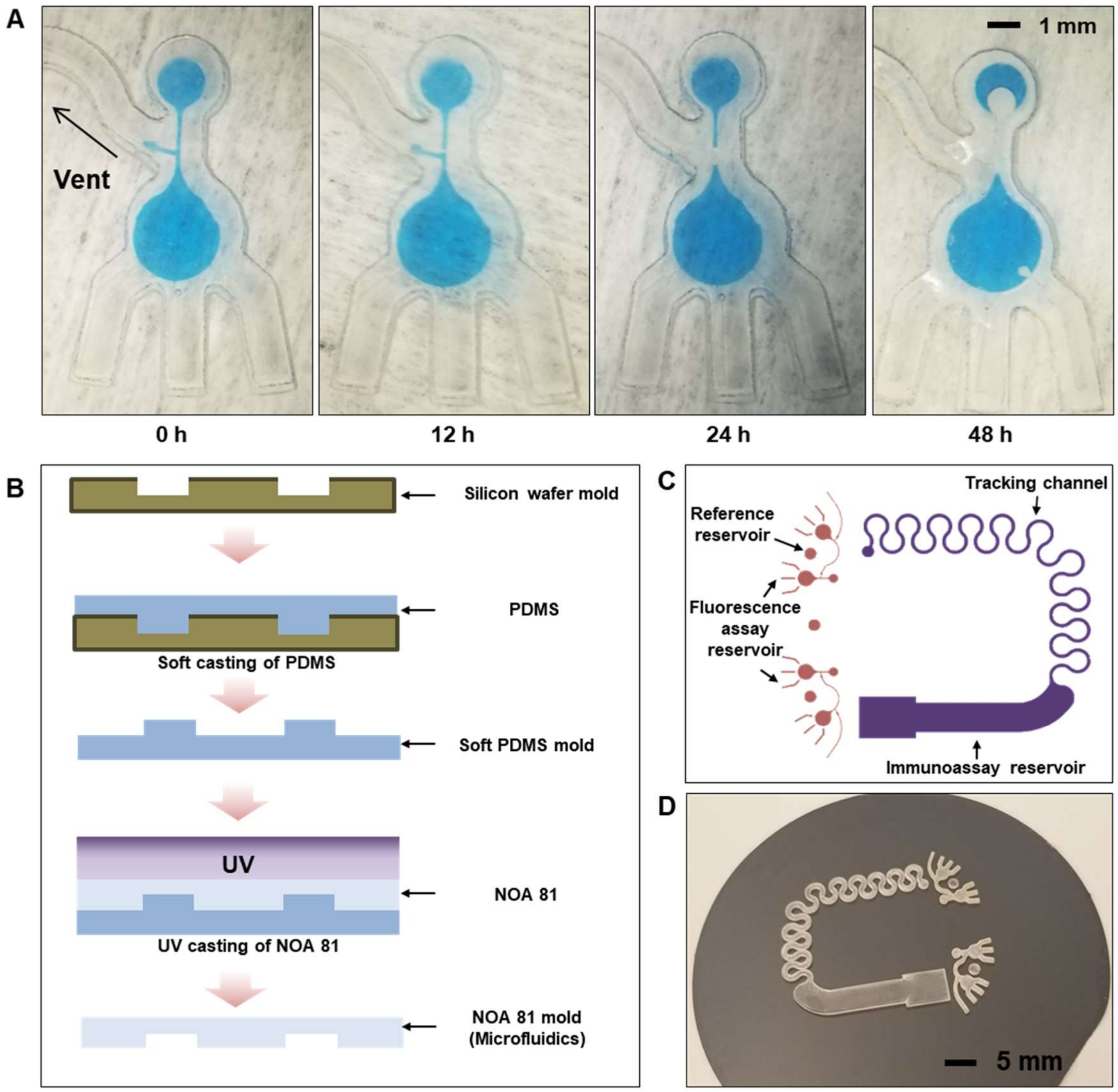

Fig. S1 Fabrication steps for the skeletal microfluidics structure. (*A*) Sequence of images that show minimal evaporation of water (blue dye) through the polyurethane material (NOA 81) used for these structures. (*B*) Steps for forming NOA81 microfluidic structures using a mold of PDMS. (*C*) Design of the skeletal microfluidics. (*D*) Optical image of the skeletal microfluidics.

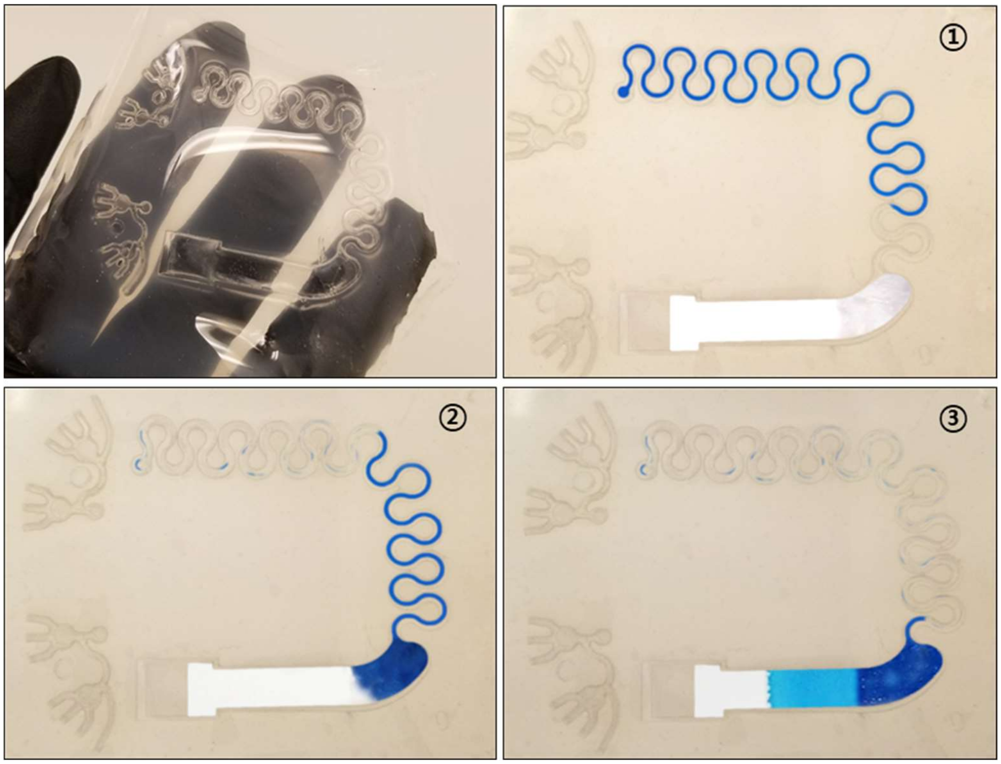

Fig. S2 Completed skeletal microfluidics and time sequential filling of microfluidics channel. Optical images of skeletal microfluidic structure with an embedded lateral flow strip filling in time (snapshots 1-3).

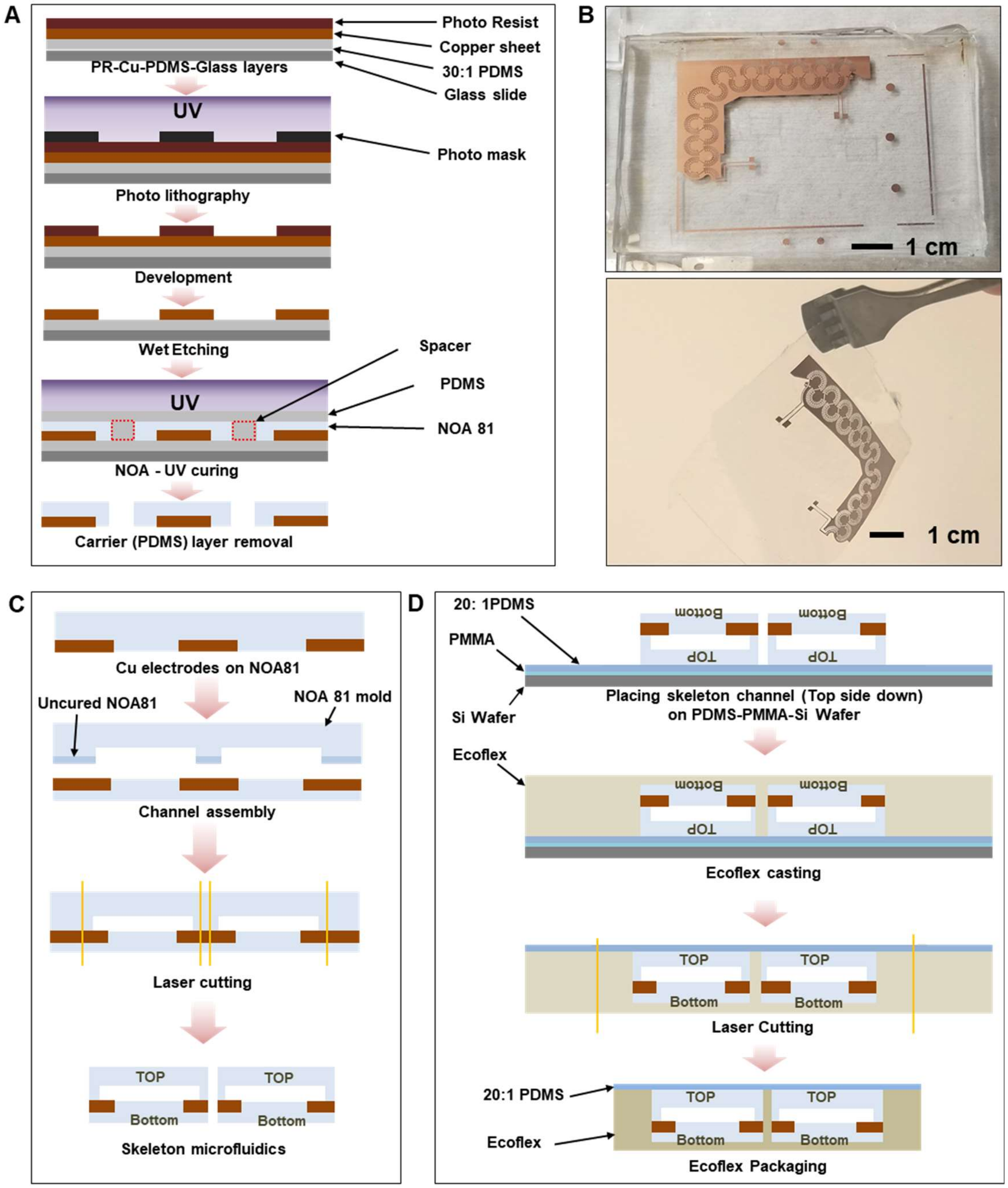

Fig. S3 Process for fabricating thin Cu electrodes on NOA81 and integration with a skeletal microfluidic structure. (*A*) Process for fabricating thin flexible Cu electrodes on NOA81. (*B*) Optical image of the fabricated electrodes. (*C*) Assembly of electrodes and an NOA81 microfluidic structure. (*D*) Process for encapsulating the skeletal microfluidic structure with Ecoflex.

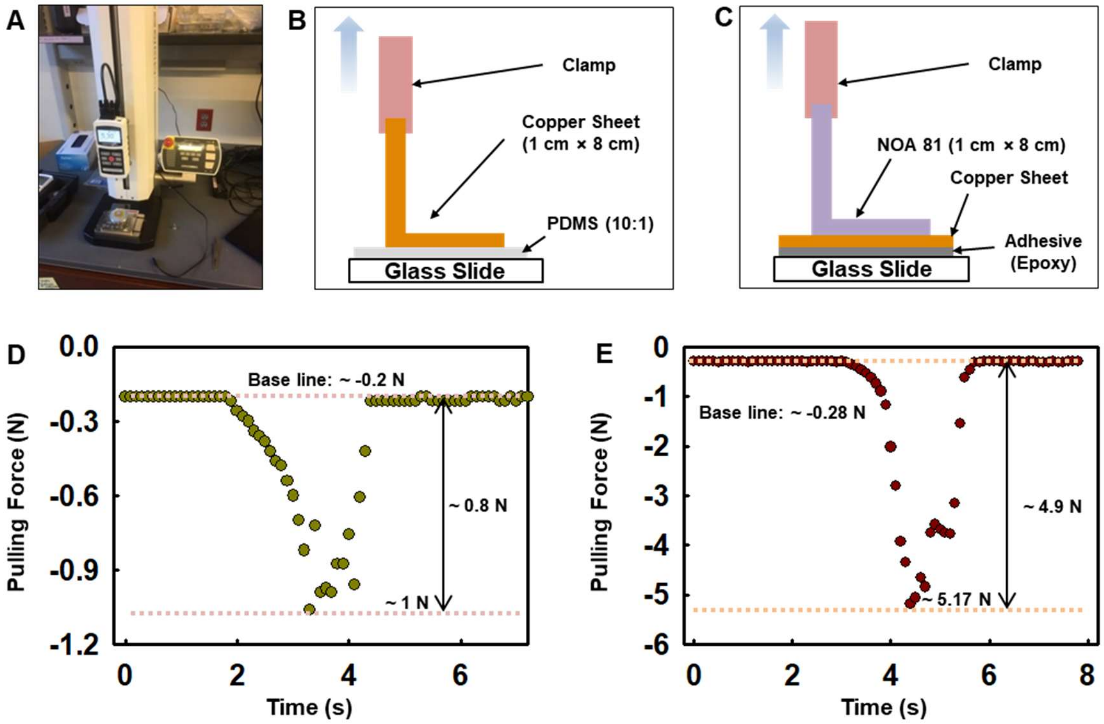

Fig. S4 Mechanical peel tests on electrode structures. (*A*) Optical image of the mechanical tester. (*B*, *C*) Schematic illustrations of the peel tests for Cu-PDMS and Cu-NOA81. (*D*) Results of peel tests between Cu and PDMS. (*E*) Results of peel tests between Cu and NOA81.

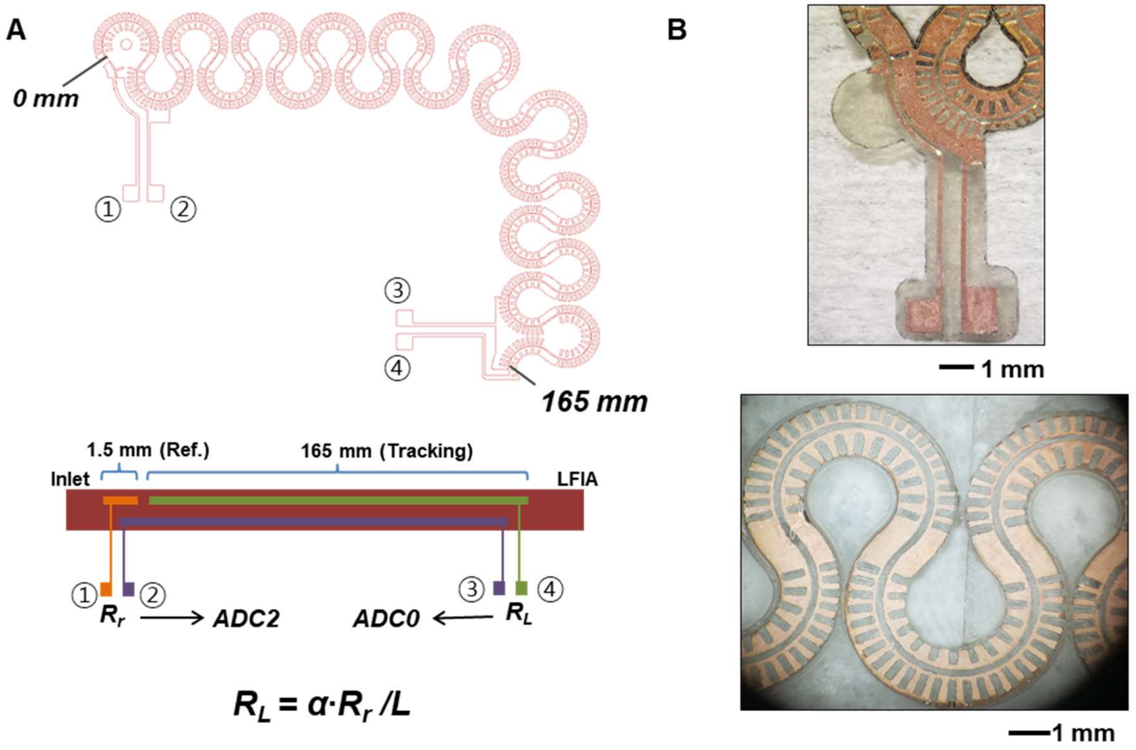

Fig. S5 Systems with integrated electrodes. (*A*) Design of serpentine and continuous tracking electrodes (top) and simplified electrodes (bottom) to highlight the measurement strategy. (*B*) Magnified view of terminals for the reference and counter electrodes (top) and serpentine, continuous electrode designs (bottom).

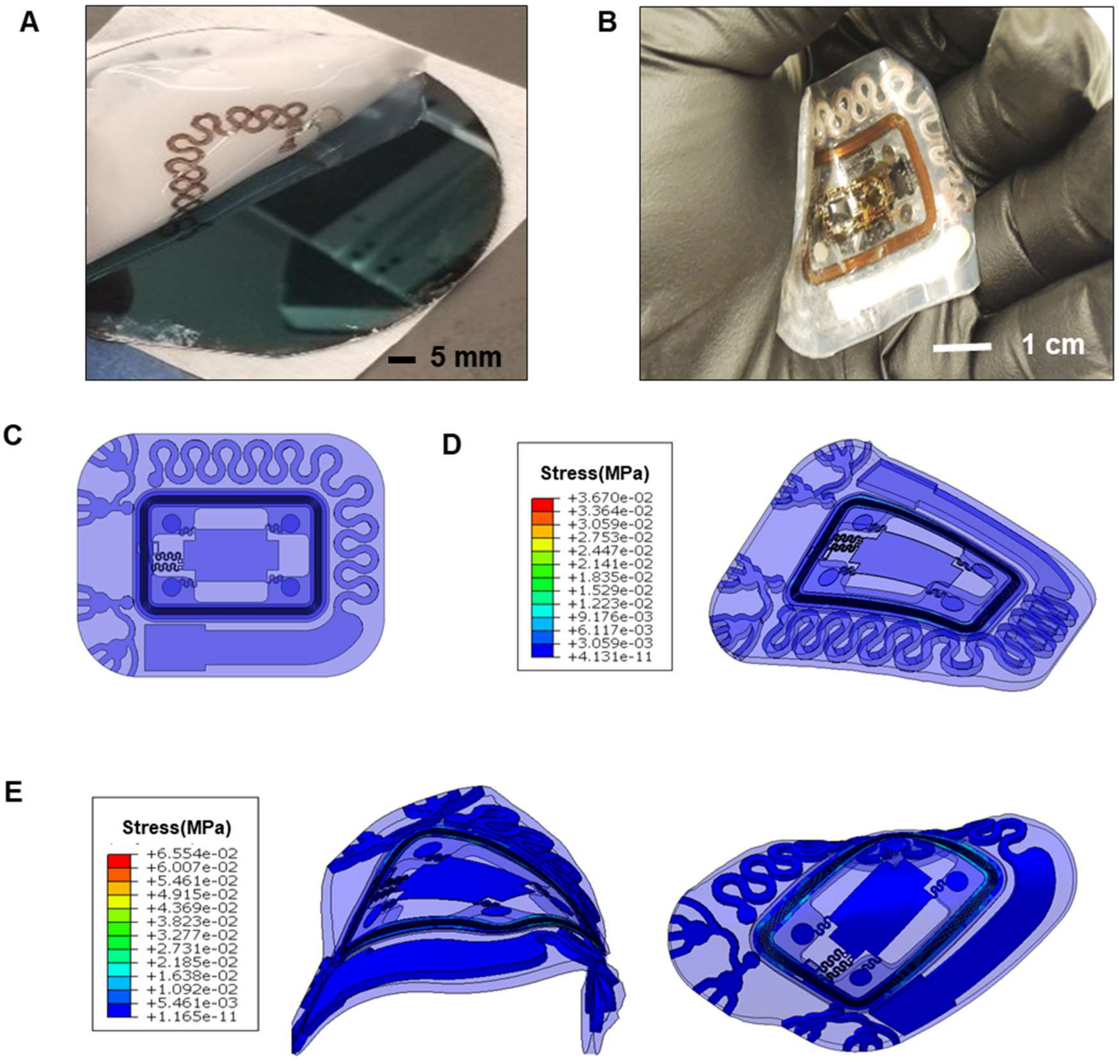

Fig. S6 Tests of the flexibility of the devices. (*A*) Optical image of skeletal microfluidic structures encapsulated in Ecoflex and peeled of a wafer. (*B*) Optical image of skeletal microfluidic device bending in the palm of a hand. (*C*) 3D mechanical simulation of device at rest. (*D*) 3D mechanical simulation during twisting. (*E*) 3D mechanical simulation during bending.

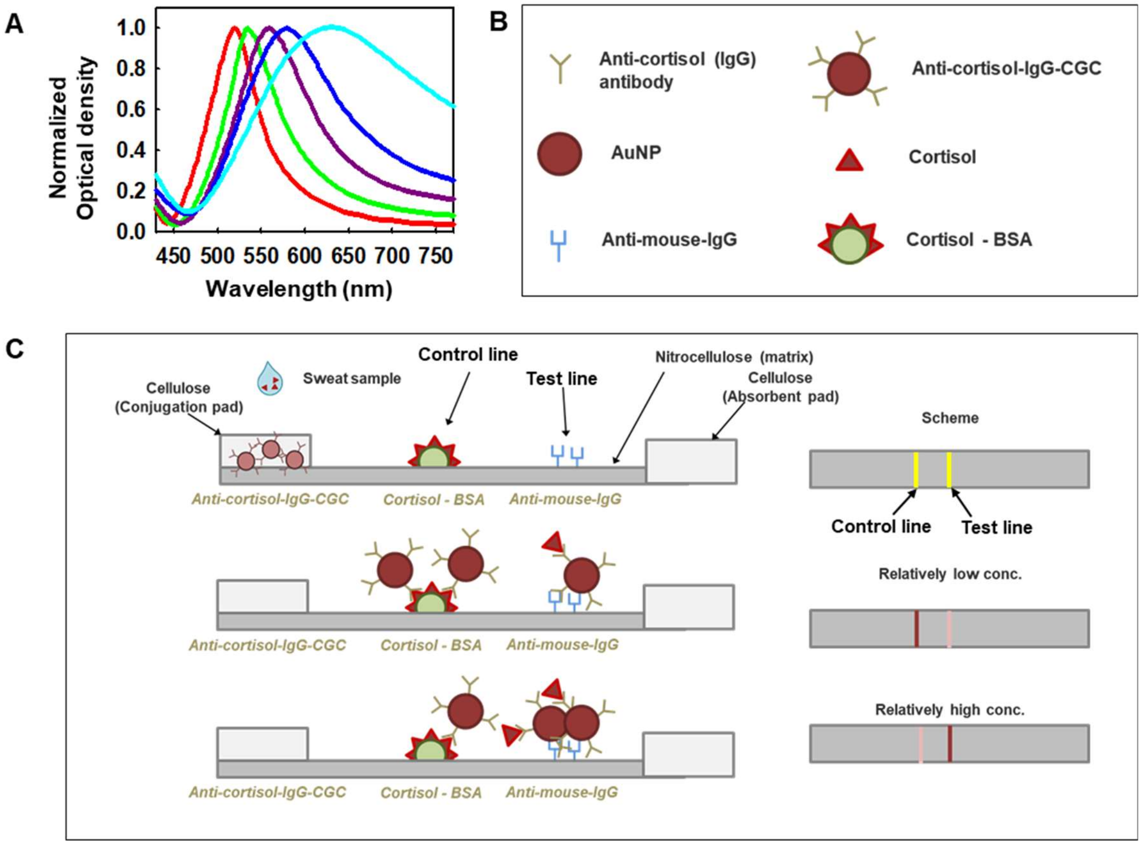

Fig. S7 Schematic illustrations of the lateral flow immunoassay with modified AuNPs. (*A*) Optical density spectrum (450-750 nm) for various sizes of AuNPs (10, 30, 50, 70 and 90 nm for red, green, brown, blue and cobalt, respectively). (*B*) Schematic symbols for the reagents in the immunoassay reaction. (*C*) Lateral flow immunoassay reactions and expected results (right).

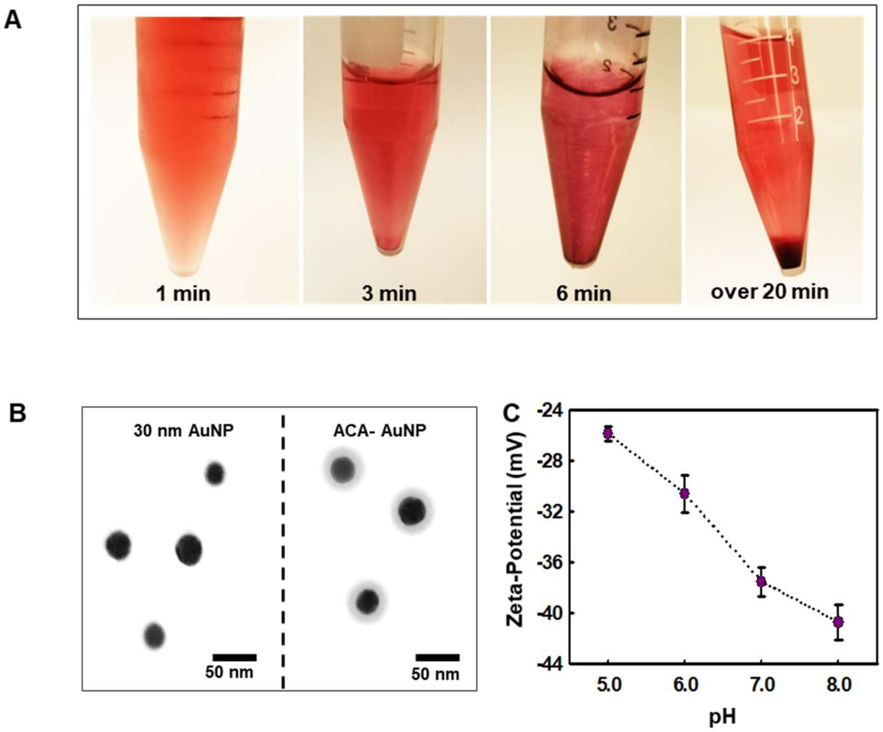

Fig. S8 Process for conjugating ACA onto AuNPs. (*A*) Optical images to show ACA-AuNP colors development at various times: 1, 3, 6 and over 20 min. (*B*) TEM images to compare AuNPs before and after ACA conjugation. (*C*) Effects of pH on the zeta-potential of ACA-AuNP conjugation.

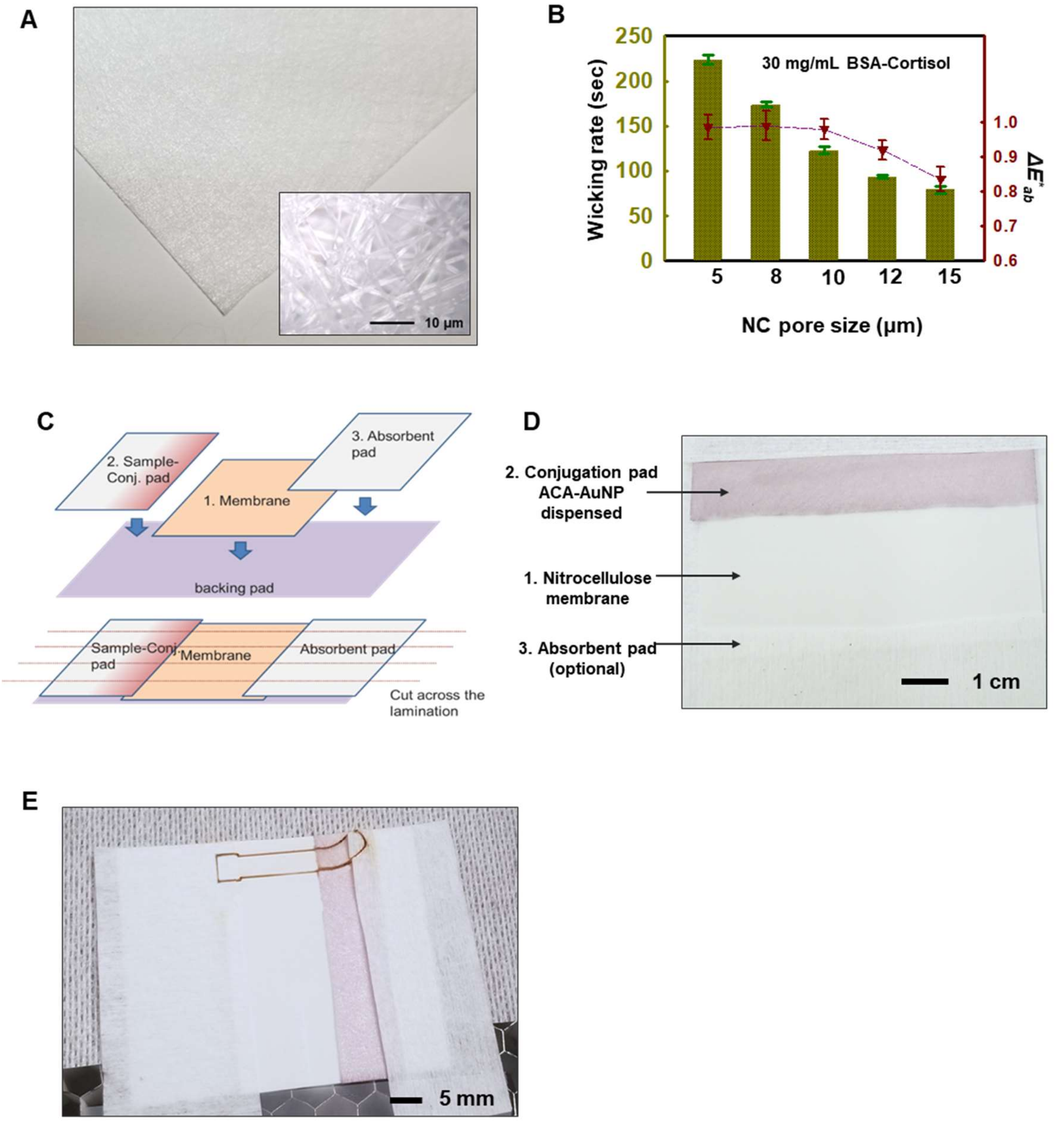

Fig. S9 Design and fabrication of the lateral flow assay platform. (*A*) Optical image of the conjugation pad and a corresponding optical microscope image (insertion). (*B*) Experimental results of wicking rate at various pore sizes of the NC membrane at 5, 8, 10, 12 and 15 μm and its effect on color development at 30 mg/mL BSA-cortisol on the NC membrane. (*C*) Schematic illustration of the lateral flow platform assembly. (*D*) Optical image after assembly of the lateral flow platform. (*E*) Optical image after laser cutting.

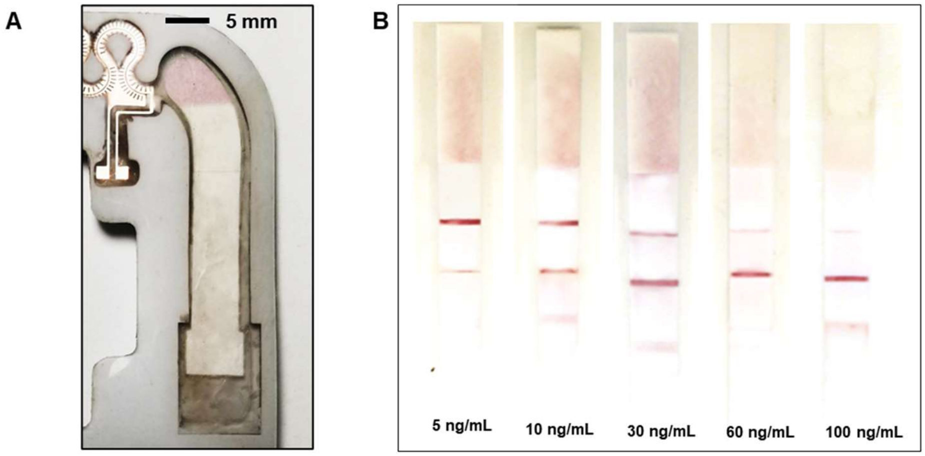

Fig. S10 Bench-top tests of the lateral flow immunoassay strip. (*A*) Assay strip embedded in the skeletal microfluidic system. (*B*) Lateral flow immunoassay tests with reagent cortisol at 5, 10, 30, 60 and 100 ng/mL after 10 min of reaction activation.

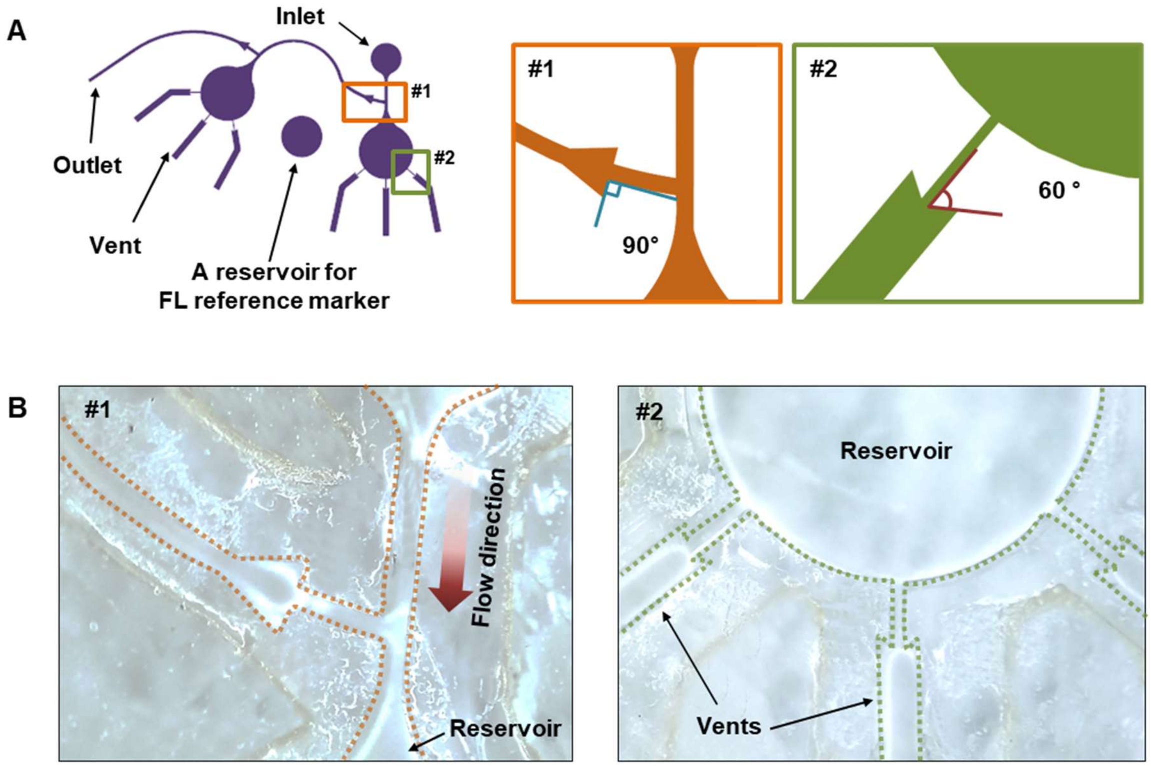

Fig. S11 Illustrations and optical images of capillary bursting valves in the microfluidic structures for the ascorbic acid and glucose assays. (*A*) Schematic drawing of the microfluidic network and β values of the valves. (*B*) Optical images of valves #1 and #2 and dashed lines along features of the valves, reservoirs and channels.

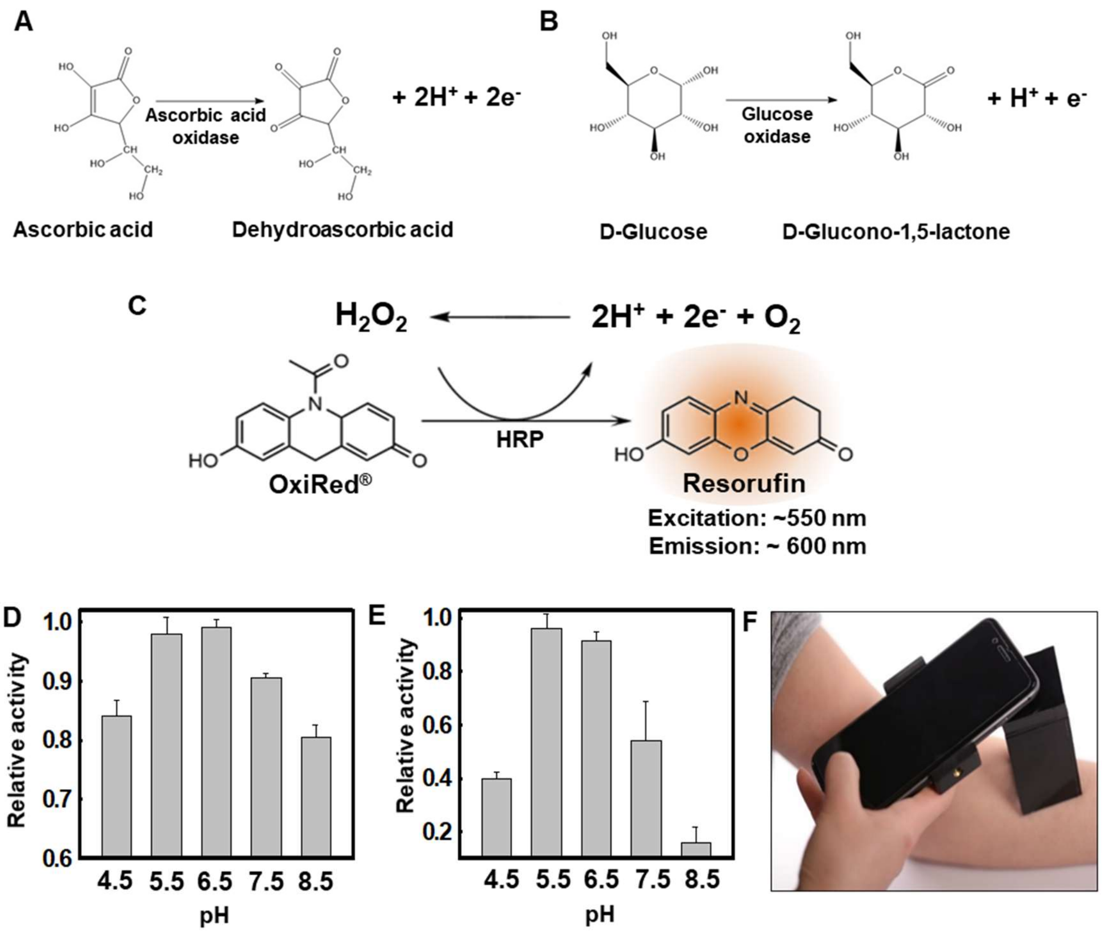

Fig. S12 Reactions and characterization of the fluorescence assays. (*A*) Ascorbic acid oxidation reaction. (*B*) Glucose oxidation reaction. (*C*) Resorufin reduction reaction. (*D*) Effects of pH on the relative activity of ascorbic acid oxidase (Three samples for each pH condition). (*E*) Effects of pH on the relative activity of glucose oxidase (Three samples for each pH condition). (*F*) Optical image of a fluorescence assay measurement with smartphone and black box apparatus.

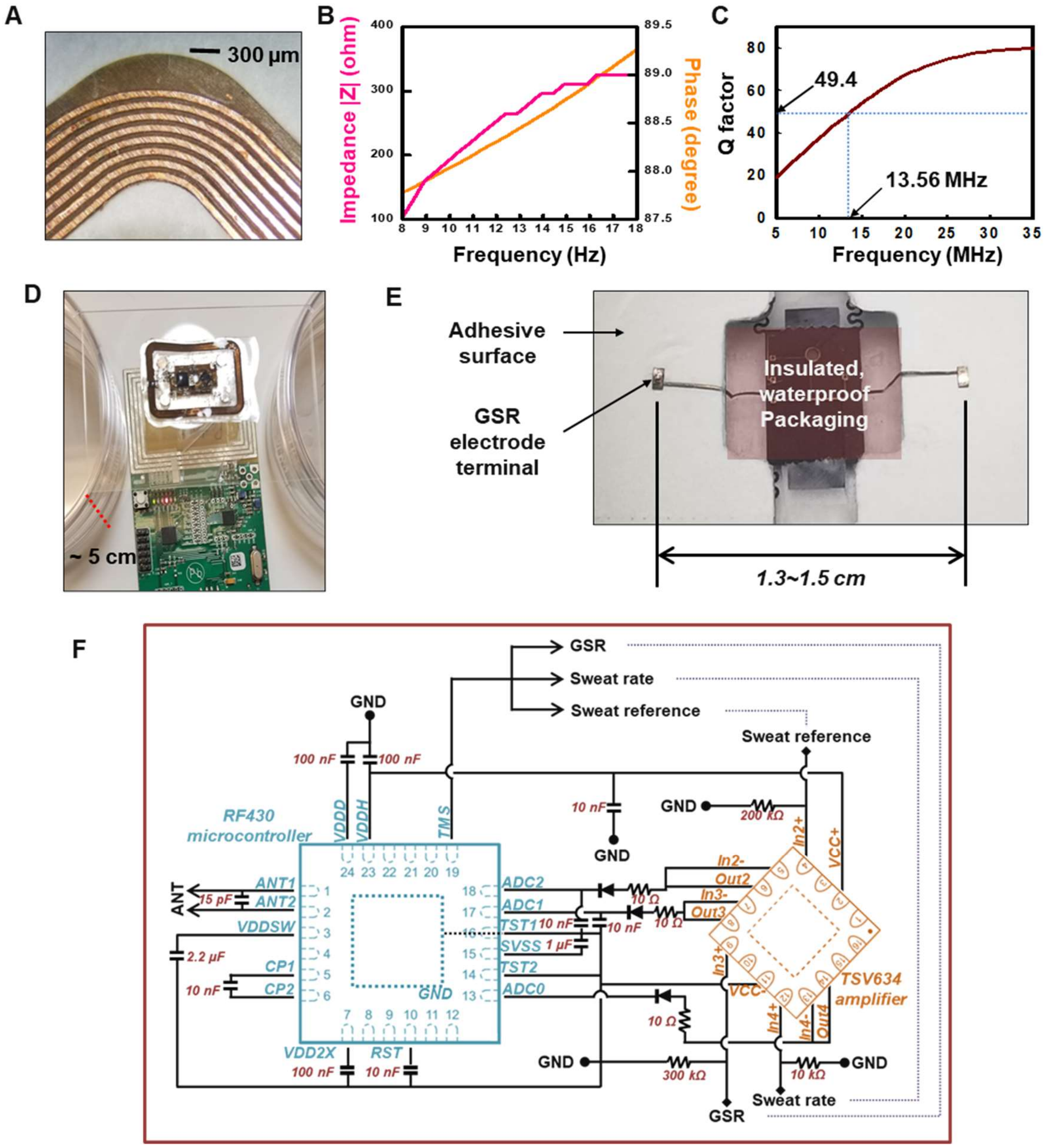

Fig. S13 Design and fabrication of the electronics and RF antenna. (*A*) Magnified optical image of the RF antenna. (*B*) Impedance and phase response of the antenna. (*C*) Measurement of the Q factor of the coil antenna. (*D*) Setup for studies of the operation distance. (*E*) Magnified optical image of GSR electrodes on the bottom of the competed device. (*F*) NFC electronics layout and chip placements.

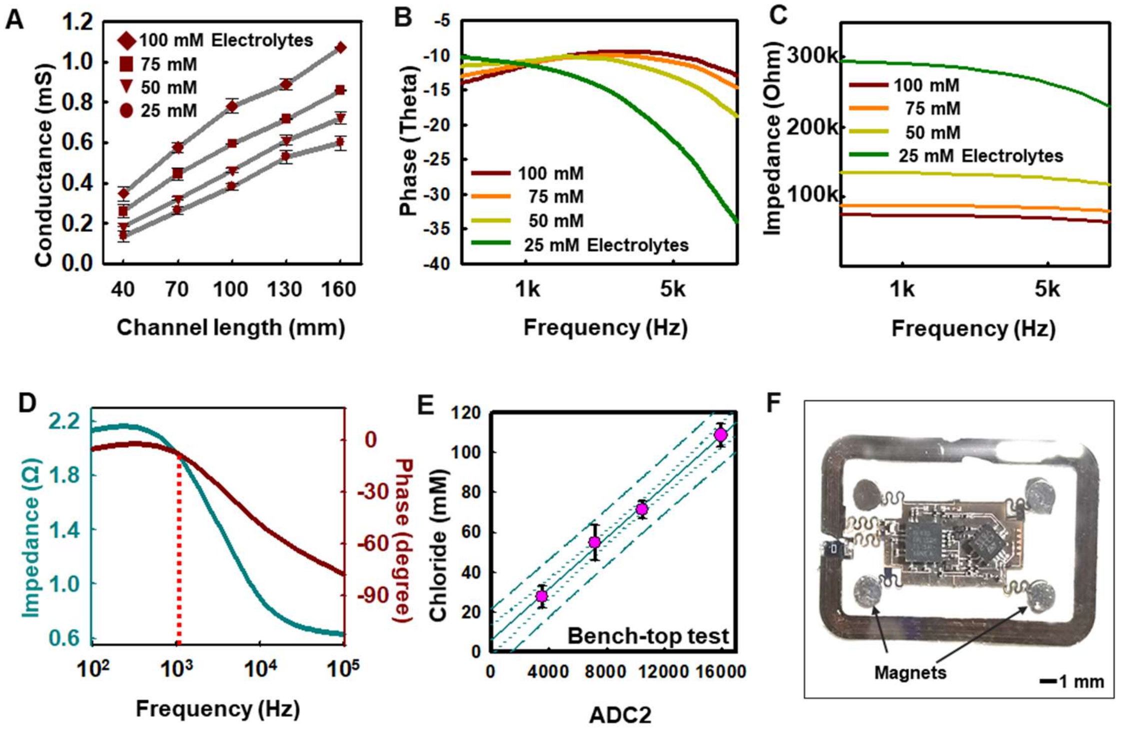

Fig. S14 Electrochemical measurements of human sweat. (*A*) Plot of the effects of electrolyte concentration on the value of ADC0 for measurement of the extent of filling of the microchannel with sweat. (*B*) Frequency dependent measurements of impedance with artificial sweat (25, 50, 75 and 100 mM). (*C*) Frequency dependent measurements of phase associated with the impedance measurement. (*D*) Fundamental measurement of skin impedance and phase. (*E*) Plot of electrolyte concentration for a series of samples of artificial sweat in the reference microchannel and corresponding ADC2 values determined by wireless readout. (*F*) Optical image of a completed electronics system packaged in PDMS and interfaced to a circular antenna.

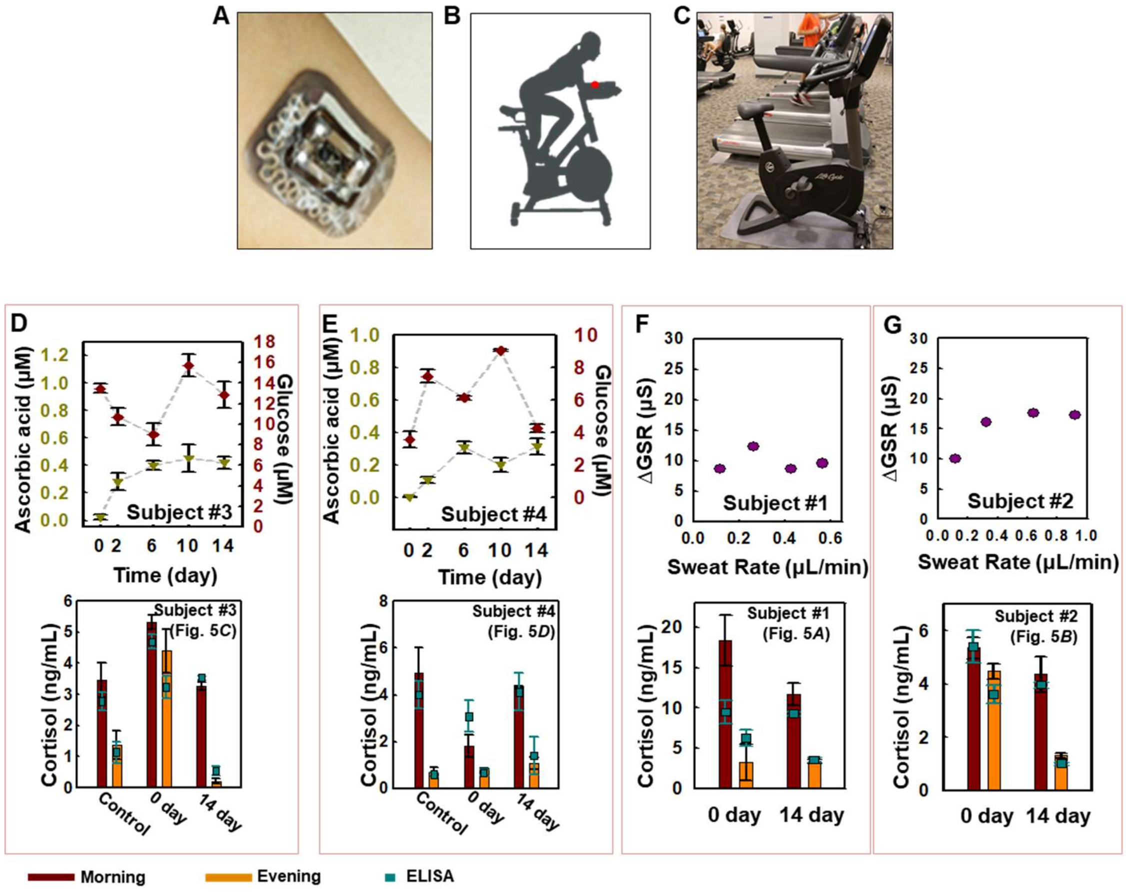

Fig. S15 Measurements of sweat ascorbic acid, glucose, and GSR during human field testing. (*A*) Optical image of the device mounted on the forearm. (*B*) Illustration of a subject on a stationary bike and location of the device. (*C*) Optical image of the stationary bike. (*D*, *E*) Daily monitoring of sweat ascorbic acid and glucose for subjects #3 and #4 at 0, 2, 6, 10 and 14 days. (*F*, *G*) Correlation of ΔGSR and sweat rate for subjects #1 and #2.

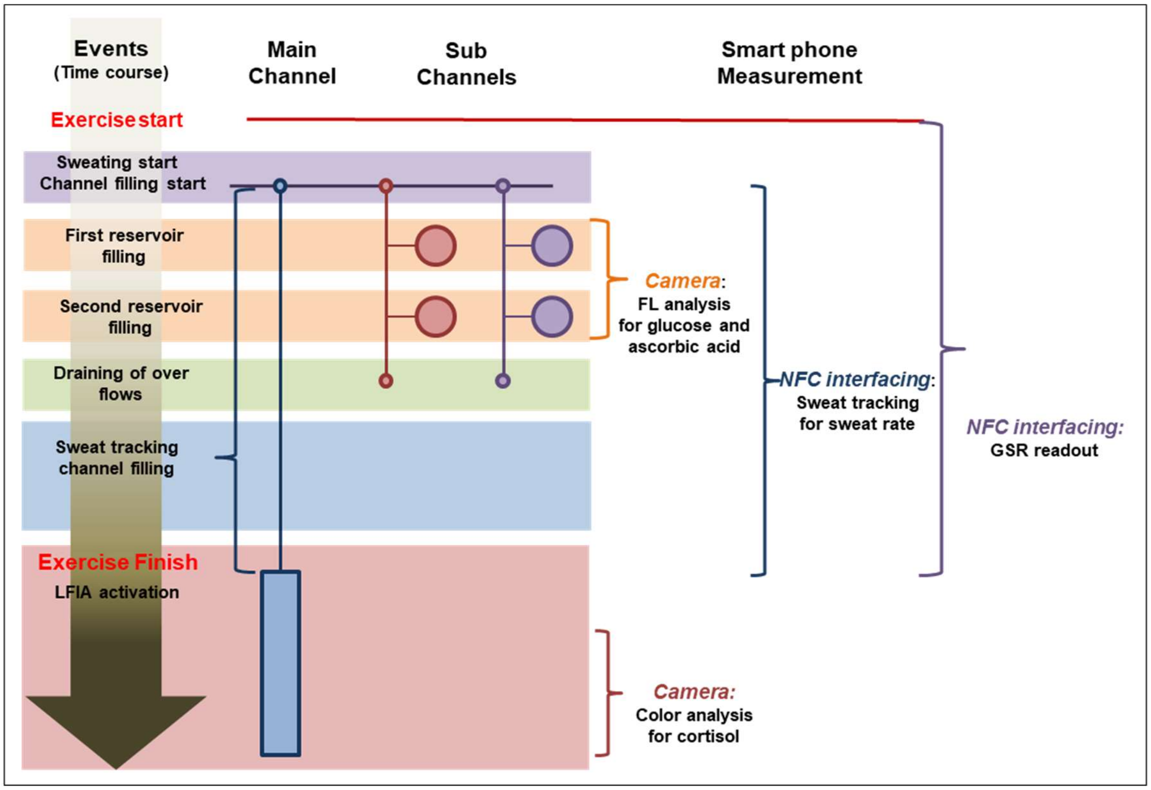

Fig. S16 Flow diagram showing sequential events as microfluidic device fills during human field testing.
